# Polyphenols in food and food wastes: Extraction, isolation, and health applications

**DOI:** 10.1016/j.fochms.2025.100351

**Published:** 2026-01-02

**Authors:** Malthe Fredsgaard, Andre Fussy, Gowri Købke Nybo, Jutta Papenbrock, Laura Sini Sofia Hulkko, Mina Dadjoo, Tanmay Chaturvedi, Mette Hedegaard Thomsen

**Affiliations:** a AAU Energy, Aalborg University, Niels Bohrs Vej 8, 6700 Esbjerg, Denmark; b Institute of Botany, Leibniz Universität Hannover, Herrenhäuser Straße 2, D-30419 Hannover, Germany; c Department of Chemistry and Bioscience, Aalborg University, Niels Bohrs Vej 8, 6700 Esbjerg, Denmark; d Haloderma, Niels Bohrs Vej 6, 6700 Esbjerg, Denmark

**Keywords:** Enzyme inhibitors, Food waste valorization, Nutraceuticals, Pharmaceuticals, Protein-polyphenol interactions, Pathogenic pathway modulation, Specialized molecules

## Abstract

Despite decades of polyphenol research, an integrated perspective on their biosynthesis, advanced extraction methods from food wastes, and potential as versatile inhibitors of pathogenic proteins and enzymes, particularly incorporating modified drug-likeness criteria, remains elusive. This integrative review compares and analyzes data on emerging polyphenol extraction and processing methods from various sources, including fruits, vegetables, berries, food production byproducts, and terrestrial sources of biomass. The drug-likeness of the reviewed polyphenols was assessed via a modified Lipinski's rule of five, and their interactions with proteins and enzymes in pathogenic pathways were investigated. The hypothesis is that polyphenols derived from food wastes exhibit high versatility as potential ligands with promising inhibitory effects that mitigate cascading disease effects in the human body. Therefore, the inhibition of proteins and enzymes involved in a wide range of diseases, including cancers, inflammatory diseases, diabetes and obesity, cardiovascular diseases, and mental and neurological disorders, was explored. Furthermore, the multifaceted nature of food and food waste-derived polyphenols was emphasized, highlighting their potential as extractable compounds with broad health-related applications. These novel insights enable targeted valorization of food wastes for personalized nutraceuticals, promote sustainable bioprocessing, and pave the way for clinical translation.

## Highlights

• SWE-NADES strategy cut organic solvents in polyphenol biorefining.
• Polyphenol diversity lessened adverse effects of high-dose single-compound supplements.
• Food waste valorization provided scalable sources for personalized nutraceuticals.
• Polyphenols showed better drug-likeness as nonselective protein ligands than synthetics.
• Challenges limited analysis of larger, glycosylated polyphenol compounds.

## 1 Introduction

Growing consumer concerns regarding the adverse effects of chemicals and synthetic additives in food, drinks, and medicine have fuelled a significant demand for natural alternatives (Rathee et al., 2023). Natural supplements can also offer numerous health benefits, including weight loss, obesity prevention, and the promotion of a healthy lifestyle, with many positive effects attributed to the consumption of polyphenols (Rana et al., 2022). In recent years, consumers have shown increased interest in enhancing their well-being through dietary choices. This trend has prompted the food and beverage industry to introduce new plant-derived additives. Options such as sugar-free, organic, calorie-free, and plant-based products provide convenient guidelines for consumers seeking healthier eating habits, aiming to minimize chemical additives and avoid overly processed foods. Consequently, consumers following this trend are likely to increase their dietary intake of phytochemicals (Ditlevsen et al., 2019). Furthermore, regulatory bodies such as the Food and Drug Administration (FDA) in the United States and the European Food Safety Authority (EFSA) in Europe actively endorse natural ingredients. These authorities are increasingly stringent in regulating food product ingridients, favoring natural alternatives over synthetically produced ones (Chauhan & Rao, 2024). This regulatory shift encourages consumers to adopt diets rich in fruits and vegetables, which are abundant in phytochemicals. Plant-synthesized specialized molecules are broadly categorized into three groups based on their chemical nature and structure: polyphenols, terpenes, and nitrogen/sulfur-containing compounds (Santos-Sánchez et al., 2019). These specialized molecules generally serve as the plant's defense system, protecting against diverse both biotic and abiotic stressors such as pathogens, insect herbivory, wounding, intense UV-B radiation, soil acidification, heavy metals, pollutants, and high salinity. They also function as nonenzymatic scavengers for reactive oxygen species (ROS) (Lopes et al., 2023; Nájar et al., 2023; Oliveira et al., 2021). Since only plants and certain microorganisms can biosynthesize polyphenols through the shikimate/phenylpropanoid pathways, these are the sole dietary sources of polyphenols for humans, or animals in general (Li et al., 2024).

Considering the substantial volume of food waste generated worldwide, global apple pomace production reaches approximately 4 million tons annually, with Germany alone contributing about 250,000 tons, most of which remains largely unutilized (Gołębiewska et al., 2022); meanwhile, European wine pomace waste is estimated at up to 14.5 million tons per year (Ratto et al., 2025). These food wastes are among the richest sources of polyphenols but are currently often composted or just used as animal feed. Instead, they can be processed into food-grade, high-concentration polyphenol dietary supplements.

High concentrations of pure polyphenols can be obtained through effective extraction and isolation methods. For example, previous research reported the isolation of polyphenols from the salt-tolerant plant species *Salicornia ramosissima* (samphire) achieving concentrations of 39.9 w% protocatechuic acid and 10.2 w% cryptochlorogenic acid. This was accomplished in just three simple, easily operated, and scalable steps, minimizing the use of organic, hazardous, and toxic solvents (Fredsgaard, Tchoumtchoua, et al., 2023).

**Fig. 1:**
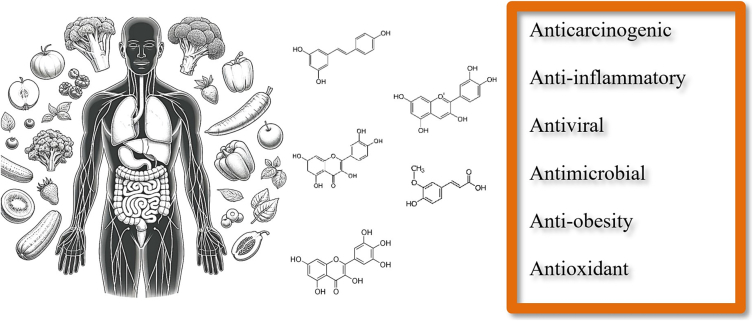
- Overview of selected health benefits of polyphenols. The image was created via OpenAI DALL·E (ChatGPT, 2025) for a graphical overview.

Polyphenols, including hydroxybenzoic acids, hydroxycinnamic acids, tannins, flavonoids, stilbenes, coumarins, and their derivatives, offer a multitude of beneficial properties. These include anti-inflammatory, antioxidant, metal-chelating, antimicrobial, anticancer, cholesterol-reducing, antiviral, and antidiabetic effects, with demonstrated efficacy against conditions such as oxidative stress, asthma, hepatitis, gastroenteritis, cardiovascular and cerebrovascular diseases, obesity and metabolic syndrome, type 2 diabetes, neurodegenerative disorders, inflammatory bowel disease, peptic ulcer disease, colorectal and other solid tumors, respiratory infections, and a range of bacterial and viral illnesses (Ahmed et al., 2025; Emran et al., 2024; Liu, An, et al., 2025; Rajha et al., 2022; Rudrapal et al., 2022; Saad et al., 2025; Singh et al., 2020) (see Fig. 1,Fig. 1 created via OpenAI DALL·E (ChatGPT, 2025) for a graphical overview).

A recently published study investigated the cognitive benefits of polyphenols, tracking 961 participants aged 60 to 100 for an average of 6.9 years through the Chicago Rush Memory and Aging Project (Holland et al., 2023). The aim was to correlate dietary habits with year over year cognitive performance. After adjusting for various factors including age, sex, educational background, cognitive and physical activity, smoking, and an Alzheimer's disease-associated protein, increased dietary intake of flavonols, specifically kaempferol and quercetin, was significantly associated with a slower rate of overall cognitive decline.

This integrative review examines and compares extraction and isolation techniques for polyphenols from food and food wastes – evaluating their cost-effectiveness, scalability, and sustainability - to harness their multi-target inhibition of pathogenic proteins and enzymes while addressing safety risks such as bioavailability limitations and toxicity for nutraceutical and pharmaceutical valorization. The motivation for writing this integrative review was the identification of knowledge gaps in both up-to-date analytical extractions methods of polyphenols, also suitable for the extraction of larger amounts, and also with respect to innovative utilisation as nutraceuticals and pharmaceutics in the light of bioeconomy.

The review is structured in the following way: After presenting the range of polyphenols as well as biosynthetic, agro-economic and molecular genetic aspects of polyphenol research that are of interest to both producers and consumers, a brief overview of the options for extraction from plant material and food waste in particular, the focus shifts to analytical methods and their strengths and weaknesses in order to subsequently highlight pharmaceutical and nutraceutical applications and the current state of knowledge about mechanisms of action. A small number of classic publications were included for a better understanding of the basics, according to the proverb “Dwarfs on the shoulders of giants”, and all meaningful available studies containing new contributions to the field of analytical tools of polyphenols from food and food wastes and their application in the nutraceutical, pharmaceutical and protein inhibitor field were selected. This approach focuses on sustainability aspects and those relating to personal usability, while also providing an opportunity to reassess the risks associated with different applications.

### 1.1 Polyphenol groups

**Fig. 2:**
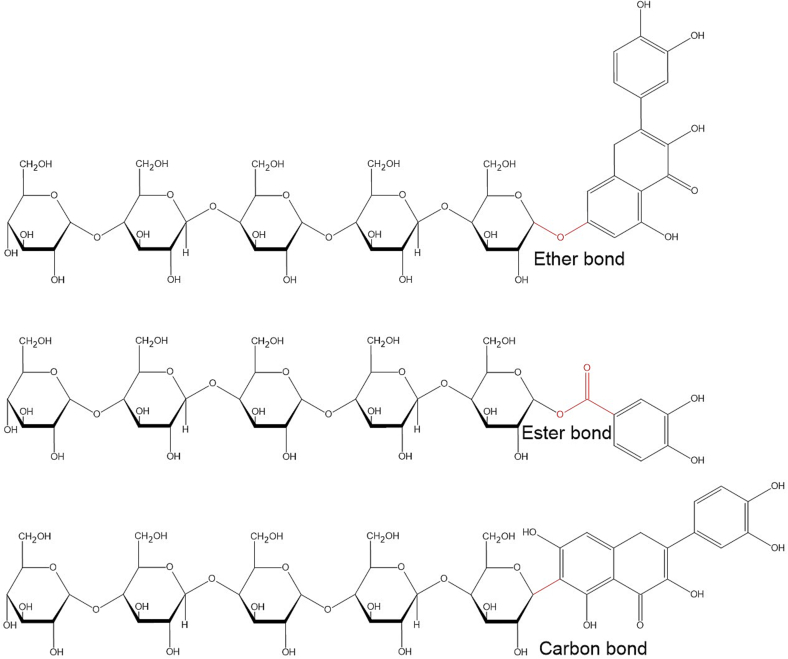
– Different bonds of polyphenols to cellulose. Quercetin is depicted with sugar chains exhibiting ether and carbon bonds, whereas the sugar chain with an ester bond is bound to protocatechuic acid. Adapted and modified graphic from Wang et al. (2020b).

There are many thousand known polyphenols in food and plant biomass – over 8000 documented compounds - existing in diverse structural forms including dimers, esters, ethers, phenolic and phenol carboxylic acids, prenylated and glycosylated conjugates, and polymerized structures (Saad et al., 2025). Polyphenols are a vast array of polar and nonpolar compounds, varying in size from >100 Da in dicarboxylic acids to approximately 30,000 Da in complex tannins (Carecho et al., 2021; Serrano et al., 2009). Only a small fraction of the total amount of polyphenols has been mapped and characterized. The structural and hydrophobic diversity among polyphenols, however, makes the detection and quantification of derivatives challenging. Polyphenols can be found as free and bound (ionic, ether, ester, and carbon–carbon) to cellulose, see Fig. 2 (adapted and modified graphic (Wang, Li, et al., 2020)), or as a link between hemicellulose and lignin (Wang et al., 2020b).

### 1.2 Hydroxybenzoic acids

Hydroxybenzoic acids are a small group of polyphenols, derived from benzoic acid with one or more hydroxyl substituents on the benzene ring. These compounds exhibit radical scavenging effects and play essential roles in reducing metal ions and chelating metals. Furthermore, they possess anticancer, anti-inflammatory, and blood sugar-regulating properties Srivastava et al., 2019). As these polyphenols are simple in structure, hydroxybenzoic acids can be produced microbially, which means that their extraction and isolation do not rely on terrestrial biomass (Li et al., 2024).

### 1.3 Hydroxycinnamic acids

Other polyphenols include hydroxycinnamic acids (HCAs) and their derivatives, which are often found in relatively high concentrations in fruits, vegetables, whole grains, cereals, coffee, tea, and wine (Sova & Saso, 2020). Like hydroxybenzoic acids, HCAs are phenolic acids with structures based on phenols featuring one or more hydroxyl substitutions. However, HCAs distinguish themselves by having an aliphatic chain connected to both the phenol and the carboxyl groups. These compounds can be covalently bound to oligosaccharides, lignin, or polysaccharides within plant cell walls. This binding explains their prevalence in lignocellulosic foods such as whole grains and cereals (Chandrakanth et al., 2022; Khan et al., 2024). HCAs and their derivatives exhibit potent antioxidative properties, making them valuable in various applications, including food packing, pharmaceuticals, cosmetics, and herbal medicine (Contardi et al., 2021). Furthermore, similar to hydroxybenzoic acids, HCAs have been shown to be microbially produced (Kumar et al., 2025).

### 1.4 Tannins

Tannins are categorized by size, physical and chemical properties, and molecular structure. They range in size from 500 to 30,000 Da and are primarily classified into two main groups: hydrolyzable tannins and condensed tannins (Serrano et al., 2009). Hydrolyzable tannins feature a central glucose molecule esterified with phenolic acids, such as gallic acid in tannic acid. In contrast, condensed tannins are a class of flavonoid polymers, characterized by carbon‑carbon bonds (C—C bonds) that are resistant to hydrolytic cleavage (Eckardt et al., 2023). Tannins possess a variety of bioactive properties, including antihypertensive activity, photoprotection, and anti-inflammatory effects. They also play an active role in chelating metals (Srivastava et al., 2019).

### 1.5 Flavonoids

Flavonoids are abundant polyphenols found in various types of biomass, with over 9000 identified throughout the plant kingdom (Dias et al., 2021; Limongelli et al., 2022). The regulation and bioavailability of flavonoids, and polyphenols in general, are influenced by both biotic and abiotic stressors. Previous studies have explored these stressors, including varying salinities, photoperiods, soilless cultivation, and differences between domesticated and wild populations (Dias et al., 2021; Oliveira-Alves et al., 2023). Additionally, flavonoids are vital in plant pollination (Jiang & Grotewold, 2025). These compounds can be categorized into eight classes, each with numerous variations and derivatives: flavanols, flavones, isoflavones, flavonols, isoflavanes, flavanones, anthocyanins, and chalcones (Dias et al., 2021; Šamec et al., 2021).

**Fig. 3:**
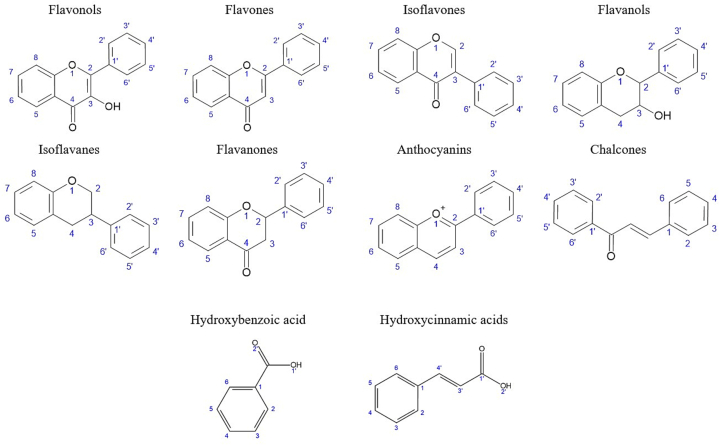
– Graphical representation of selected polyphenol structures. See supplementary material Table A.10 for the structural descriptions of flavanols, flavones, isoflavones, flavonols, isoflavanes, flavanones, anthocyanins, chalcones, hydroxybenzoic acids and hydroxycinnamic acids presented in this review.

For a graphical representation of selected polyphenol structures, please refer to Fig. 3.

## 2 Development of biomarkers for optimal polyphenol biomass cultivation

### 2.1 Biosynthesis of polyphenols

The biosynthesis process of polyphenols begins with the shikimate/phenylpropanoid pathway, which is initiated by the condensation of phosphoenolpyruvate with erythrose-4-phosphate. This pathway produces many monomeric and polymeric polyphenolic specialized molecules in plants. It culminates in the production of chorismate, a precursor to tryptophan and phenylalanine/tyrosine biosynthesis. As the first step in the phenylpropanoid pathway, phenylalanine is converted into cinnamic acid by phenylalanine ammonia-lyase (PAL). Cinnamic acid is subsequently hydroxylated to *p*-coumaric acid by cinnamate 4-hydroxylase (C4H). A branch of this pathway generates *p*-coumaric acid indirectly from tyrosine via tyrosine ammonia lyase (TAL). This alternative route, which is more common in microorganisms, circumvents the need for the P450 enzyme cinnamate 4-hydroxylase in the phenylpropanoid pathway. Depending on the pathway's branching, the intermediate *p*-coumaric acid can be further transformed into lignans, coumarins, or other HCAs. In the phenylpropanoid pathway, *p*-coumaric acid is further converted into 4-coumaroyl-CoA via a process mediated by *p*-coumaric acid-CoA ligase (4CL). This compound serves as a precursor for biomass-specific compounds such as stilbenes, flavonoids, and condensed tannins. While the shikimate- and phenylpropanoid pathways supply *p*-coumaroyl-CoA, the polyketide pathway mediates C2 elongation via the use of malonyl-CoA as the condensing unit (Shende et al., 2024).

In the synthesis of various complex polyphenols, including chalcones, flavones, flavonols, and isoflavones, the key enzymes identified are chalcone synthase, chalcone reductase, chalcone isomerase, isoflavone synthase, flavanone 3-hydroxylase, dihydroflavonol reductase, flavonol synthase, isoflavone hydroxylase, and isoflavone reductase (Mao et al., 2025). Flavonoids are synthesized through a combination of the phenylpropanoid and polyketide pathways. Here, C6-C3 phenyl-propanoic intermediates are metabolized, and the polyketide units are linked and modified through various enzymatic steps within the flavonoid biosynthesis pathwaydia (Dias et al., 2021; Mao et al., 2025).

**Fig. 4:**
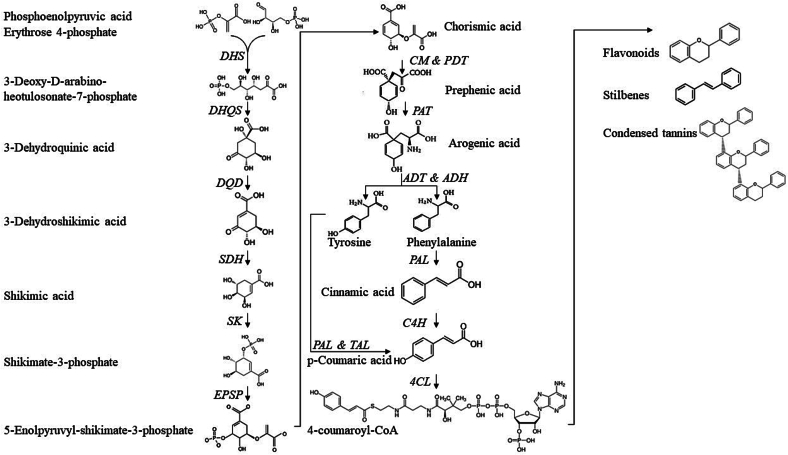
– Shikimate and phenylpropanoid pathways expressed in plants. Molecules are shown in their protonated states. Enzymes are given in italics. The figure is adapted from previous research (Ninkuu et al., 2025; Shende et al., 2024). DHS: 3-Deoxy-D-arabino-heotulosonate-7-phosphate synthase, DHQS: 3-Dehydroquianate synthase, DQD: 3-Dehydroquianate dehydratase, SDH: Shikimate 5-dehydrogenase, SK: Shikimate kinase, EPSP: 5-Enolpyruvyl-shikimate-3-phosphate synthase, CM: Chorismate mutase, PDT: Prephenate dehydratase, PAT: Prephenate aminotransferase, ADT: Arogenate dehydratase, ADH: Arogenate dehydrogenase, PAL: Phenylalanine ammonia-lyase, C4H: Cinnamate 4-hydroxylase, TAL: Tyrosine ammonia-lyase, 4CL: 4-Coumarate:CoA ligase.

See Fig. 4 for a graphical representation of the shikimate and phenylpropanoid pathways leading to the synthesis of flavonoids, stilbenes, and condensed tannins (adapted from previous research (Ninkuu et al., 2025; Shende et al., 2024)).

In certain plant species, modifications of these pathways can be observed. For example, concerning lignin biosynthesis, members of the Poaceae family bypass the cinnamate hydroxylation step, which is typically mediated by the membrane-bound cytochrome P450 C4H. They achieved this by generating coumarate directly from tyrosine via the bifunctional *L*-phenylalanine/L-tyrosine ammonia-lyase (PTAL). This capability is linked to the distinctive composition of grass cell walls. Coumarate is subsequently converted to caffeate by bifunctional coumarate 3-hydroxylase (C3H)/ascorbate peroxidase (APX), which is then further transformed into ferulate by caffeic acid 3-*O*-methyltransferase (COMT). Both caffeate and ferulate intermediates are essential precursors for lignin biosynthesis (Barros & Dixon, 2020). In the genus *Salvia* (Lamiaceae), most polyphenols are produced through the phenylpropanoid pathway. The common precursors are phenylalanine and tyrosine, and their biosynthetic pathways involve two parallel branches with five rate-limiting enzymes: PAL, C4H, 4-coumarate: CoA ligase, TAL, and rosmarinic acid synthase (Wang, Shan, et al., 2019). These findings underscore the importance of identifying and characterizing the key biosynthetic pathways for each taxon to determine specific biomarkers.

### 2.2 Pedoclimatic and agronomic factors that increase the amount of polyphenols

For biorefinery applications, the amount of polyphenols per kilogram of biomass is economically crucial, with higher contents leading to a more efficient extraction process. The polyphenol content and composition typically vary with a plant's developmental stage and specific organ. Consequently, species that are naturally rich in polyphenols are particularly relevant. An alternative strategy involves inducing polyphenol biosynthesis through environmental factors, such as the application of mild stress (Sharma et al., 2019). These environmental factors influencing polyphenol content can be broadly categorized as either pedoclimatic (related to climatic conditions) or agronomic (related to cultivation techniques).

According to a previously composed protein competition model, high protein synthesis rates lead to low polyphenol production rates. This occurs due to the competition for the amino acid phenylalanine (PHE) as a precursor (Klem et al., 2022). In line with the carbon–nitrogen balance hypothesis, the concentration of carbon-based specialized molecules, such as polyphenols, is inversely correlated with nitrogen availability. Under optimal nutrient conditions, a plant is assumed to prioritize vegetative growth by allocating resources (mainly nitrogen and carbon) to it, resulting in less carbon being available for polyphenol synthesis. In support of these findings, a previous study reported the highest total phenolic content (TPC) in basil plants grown under low nitrogen conditions. Conversely, nitrogen-based specialized molecules are predicted to be directly correlated with nitrogen availability (Liu, Dong, et al., 2025).

Light availability significantly impacts the production of most flavonoids. During ripening, the composition, concentration, and proportion of polyphenols change (Wu et al., 2025). Increased UV radiation can increase the production of UV-protective flavonoid group anthocyanins in *Brassica rapa* as a stress response, enabling plants to adapt to changing environments (Neugart & Bumke-Vogt, 2021). In addition to their role in scavenging induced ROS within mesophyll and chloroplasts – where free polyphenols are predominantly found in vacuoles and bound forms in cell walls (Bobrovskikh et al., 2020) – certain flavonoids, particularly shielding flavonoids located in the epidermis, also act as blocking agents by reflecting UV light before it reaches the chloroplasts (Dini & Grumetto, 2022). Any ROS that diffuse from chloroplasts can then be further scavenged by polyphenols in vacuoles (Bobrovskikh et al., 2020).

Under drought conditions, electron acceptors in the electron transport chain become limited, leading to an accumulation of free radicals. To neutralize these tissue-damaging compounds, the phenylalanine pathway is activated to produce polyphenols, primarily anthocyanins and other flavonoids (Dabravolski & Isayenkov, 2023). A similar protection mechanism is observed when plants are exposed to high temperatures. Various polyphenols, including anthocyanins, flavonols, tannins, and phenolic acids, are upregulated to neutralize the ROS produced. Additionally, to reduce membrane permeability and prevent water loss, the production of *p*-coumaric, ferulic, and caffeic acids is upregulated (Alhaithloul et al., 2021). The observed upregulation of polyphenols in plants grown under cold conditions can presumably be explained by the presence of the cryoprotective molecule glycine betaine, which promotes the activity of the key enzyme PAL (Wang, Xu, et al., 2019).

Like drought and heat stress, soil salinity induces photosynthetic stress, leading to the formation of ROS. As a protection mechanism, the overall polyphenol content is increased. This phenomenon has been observed in several halophyte species, including *Sesuvium porulacastrum* and *Crithmum maritimum*, *Triglochin maritima* and *Halimione portulacoides*. Additionally, certain halophytic mangrove species exhibit high polyphenol contents, such as condensed tannins (Boestfleisch & Papenbrock, 2017; Glasenapp et al., 2019). Interestingly, even in glycophytic species like the aromatic *Thymus* genus, polyphenol content increases in the presence of salt (Bistgani et al., 2019).

Not only abiotic stressors can trigger an upregulation of certain polyphenols. The phytohormone jasmonic acid, produced as a response to tissue damage from herbivores, initiates a signal cascade that results in polyphenol production (Pan et al., 2019). These compounds can reduce herbivore fitness by altering egg development and decreasing parental fertility (Tayal et al., 2020). Furthermore, not only are polyphenols found in plant biomass, they can also be secreted from the root system as root exudates. This secretion can occur in response to stressors, functioning as signaling chemicals to nearby plants and microorganisms, or as an essential component of the nodulation process in legumes (Andersen et al., 2022; Andersen et al., 2023).

Organically grown vegetables demonstrate higher total phenolic and anthocyanin content compared to conventionally cultivated counterparts, with corresponding higher antioxidant capacity. This elevated phenolic compound production reflects a stress-response mechanism, whereby the absence of synthetic pesticides and reduced nitrogen fertilization in organic systems increase pathogenic and abiotic stress, thereby activating phenylpropanoid biosynthesis and antimicrobial compound production. However, the effect of cultivation method is plant species and cultivar-dependent; in fruits and legumes, this pattern is not consistently observed, with conventional production sometimes yielding equivalent or higher phenolic content (Cruz-Carrión et al., 2023).

### 2.3 Key genes as biomarkers to determine the optimal harvesting time point

Several genes have been identified as pivotal in the central phenylpropanoid pathway, including *C4H*, ferulate 5-hydroxylase (*F5H*), cinnamoyl-CoA reductase (*CCR*), *COMT*, *PAL*, and chalcone synthase (*CHS*) (Joshi et al., 2022). The copy numbers of these key genes often vary by species. For instance, *Medicago truncatula* has six isoforms of *PAL* genes, *Populus trichocarpa* has five, and *Oryza sativa* has nine. These isoforms differ primarily in their activity and cellular localization. In *Arabidopsis thaliana,* for example, *PAL1* is primarily expressed in vascular tissues, whereas *PAL2* and *PAL4* are expressed in seeds. In addition to individual genes, certain transcription factors also play a role in inducing polyphenol biosynthesis, such as WRKY1 in *Oryza sativa*. However, these transcription factors are typically also positively associated with other metabolic pathways. A comprehensive review examining the impact of various environmental factors on polyphenol biosynthesis reported that an increase in polyphenol content was generally accompanied by increased expression of *PAL* and *CHS* (Sharma et al., 2019). Therefore, analyzing the expression of organ-specific isoforms of these key genes could serve as biomarkers for conditions that induce polyphenol biosynthesis. Importantly, only mild stress conditions are advisable, as severe stress can decrease biomass yield due to growth inhibition, thereby lowering the total amount of polyphenols extractable per unit area (Boestfleisch & Papenbrock, 2017).

## 3 Extraction methods

The access of polyphenols found in plant cell vacuoles requires physical treatment to break the plant cell walls (Chaturvedi et al., 2022; Tzima et al., 2021). Extraction methods must selectively fractionate and solubilize target polyphenols from lignocellulosic biomass; however, excessive biomass deconstruction introduces undesirable complications. One critical concern is the formation of toxic furan compounds - particularly furfural from C5 sugars (pentoses from hemicellulose) and 5-hydroxymethylfurfural (HMF) from C6 sugars (glucose from cellulose) - resulting from acid-catalyzed thermal degradation of cellulose, hemicellulose, and other polysaccharides. The extent of furan formation serves as a severity indicator of biomass deconstruction during extraction pretreatment, correlating directly with unintended depolymerization of the lignocellulosic network and thus compromising both extraction selectivity and product purity (Moron et al., 2023). Furthermore, fermentation inhibitors are generated during hydrothermal pretreatment and include furyl compounds (furfural and hydroxymethylfurfural [HMF]) and phenolic compounds (vanillin, vanillic acid, and related polyphenols). Regarding bioethanol production these degradation products significantly reduce fermentation efficiency and inhibit yeast growth and ethanol production (Greetham et al., 2019). In a another review, the authors described different extraction methods of bound polyphenols from food plants. Chemical methods for extracting bound polyphenols include acid or alkali treatment, which can hydrolyze and degrade polyphenols into smaller phenolic compounds. These treatments sometimes involve complex pretreatments, requiring inert atmospheres (e.g., argon or nitrogen gas) and the addition of antioxidants (e.g., EDTA or ascorbic acid) to prevent polyphenol oxidation (Wang, Li, et al., 2020).

### 3.1 Deep eutectic solvents

Deep eutectic solvents (DES) are mixtures of two or more compounds – typically a hydrogen bond donor (HBD) and hydrogen bond acceptor (HBA) - that form eutectic mixtures with substantially lower melting points than individual components (Hikmawanti et al., 2021). Natural deep eutectic solvents (NADES) consist of biodegradable, biocompatible compounds such as choline chloride (ChCl), sugars, organic acids, amino acids, and polyols combined in defined molar ratios for specific applications (Coscarella et al., 2023). Choline chloride-based DES formulations exhibit exceptional selectivity for polyphenols of diverse polarities through supramolecular hydrogen bonding interactions that stabilize phenolic hydroxyl and carbonyl groups, demonstrating superior extraction performance compared to conventional organic solvents (Islamčević Razboršek et al., 2020).

The physicochemical properties of DES are tailored by modulating HBA and HBD composition and molar ratios. Higher HBD polarity and lower water content correlate with increased total anthocyanin recovery from grape pomace, with more acidic HBD systems (such as oxalic acid-based formulations) exhibiting superior extraction efficiency compared to less polar alternatives through pH and polarity effects on acid-labile compound stabilization (Errichiello et al., 2025).

A decisive advantage of DES-mediated extraction is the dual functionality of polyphenol-rich extracts. NADES extracts demonstrated 10-fold higher antimicrobial activity against *Bacillus subtilis* and *Bacillus cereus* compared to ethanolic and aqueous extracts. This enhanced multifunctional bioactivity – combining antioxidant and antimicrobial properties - eliminates the need for further polyphenol purification in food ingredient applications. However, this dual-effect phenomenon results from both polyphenol content and inherent antimicrobial properties of DES components (ChCl, betaine, propylene glycol), complicating standardization and regulatory approval for pharmaceutical formulations requiring specific bioactivity attribution (García-Roldán et al., 2022).

Table 1 provides an overview of the relevant literature applying DES solvent extraction to recover polyphenols from various types of biomass.

**Table 1:** – Literature review of extraction of polyphenols using deep eutectic solvents. HBA: Hydrogen bond acceptor. HBD: Hydrogen bond donor. MAE: Microwave-assisted extraction. MA: Maceration. UAE: Ultrasound-assisted extraction. ChCl: Choline chloride. LA: Lactic acid. ND: No data.

**Botanical name**	**HBA**	**HBD**	**Solid-to-solvent** **ratio(w/v)**	**Molar ratio** **(HBA:HBD:H**_**2**_**O)**	**Time** **(min)**	**Temperature** **(°C)**	**Extraction** **method**	**Extracted polyphenols**	**Ref.**
*Myrciaria cauliflora*	ChCl	Propylene glycol	1:30	1:1:2	60	50	MA	Kuromanin	(Benvenutti et al., 2020)
*Olea europaea* oil	ChCl	Xylitol	1:1	2:1:3	60	40	MA	Hydroxytyrosol, tyrosol, oleacein, oleocanthal, luteolin, apigenin, pinoresinol, 1-acetoxypinoresinol	(Rodríguez-Juan et al., 2021)
*Camellia oleifera* seed oil	ChCl	Glycerol	1:1	1:2:0	60	50	MA	Gallic acid, *p*-hydroxybenzoic acid, protocatechuic acid, benzoic acid, phthalic acid, *p*-hydroxyphenylacetic acid, vanillic acid, cinnamic acid, *p*-coumaric acid, caffeic acid, ferulic acid, sinapic acid, chlorogenic acid, apigenin, kaempferol, naringenin, luteolin, quercetin, myricetin, vitexin, taxifolin, catechin, epicatechin, epigallocatechin gallate, epigallocatechin	(Wang, Jia, et al., 2020)
*Prunus ceranus* pomace	ChCl	Malic acid	3:40	1:1 and 20 % (*w*/w)	0.5	ND	MAE	Cyanidin-3-sophoroside, cyanidin-3-glucosylrutinoside, keracyanin, quercitrin, rutin, isoquercitrin, narcissin	(Popovic et al., 2022)
*Ilex paraguariensis* leaves	Lactic acid	Glycerol	1:20	3:1:3	60	40	UAE	Chlorogenic acid, rutin, ferulic acid	(Rebocho et al., 2022)
*Thymus serpyllum*	L-proline	Glycerol	1:28	1:2:1	180	65	MA	Gallic acid, caffeic acid, epicatechin, rosmarinic acid, luteolin, quercetin	(Pavlić et al., 2022)
*Coriandrum sativum* seeds	ChCl	Urea	1:20	1:1 and 40 % (*w*/w)	30	25	UAE	Chlorogenic acids, protocatechuic acid, caffeic acid, *p*-coumaric acid, rutin	(Ianni et al., 2023)
*Coriandrum sativum* seeds	ChCl	Glucose	1:20	1:1 and 40 % (w/w)	30	25	UAE	Protocatechuic acid, caffeic acid, *p*-coumaric acid, chlorogenic acids, rutin	(Ianni et al., 2023)
*Coriandrum sativum* seeds	ChCl	Citric acid	1:20	1:1 and 40 % (w/w)	30	25	UAE	Rutin, protocatechuic acid, caffeic acid, *p*-coumaric acid, chlorogenic acids	(Ianni et al., 2023)
*Lycium barbarum*	ChCl	*p*-Toluenesulfonic acid	1:200	1:2:0	90	25	UAE	Chlorogenic acid, morin, luteolin, *p*-coumaric acid, ferulic acid, hyperoside, rutin, myricetin, quercitrin, apigenin	(Ali et al., 2019)
*Mangifera indica* peel and seeds	ChCl	Glycerol	1:60	1:2:0	5	25	UAE	Gallic acid, catechin, mangiferin, hyperoside, ellagic acid	(Ojeda et al., 2023)
*Rosmarinus officinalis*	ChCl	1,2-propanediol	1:40	1:2 and 50 % (w/w)	150	65	MA	Carnosic acid, carnosol, rosamric acid	(Wojeicchowski et al., 2021)
*Aegle marmelos*	ChCl	Oxalic acid	1:50	1:1 and 25 % (*v*/v)	60	80	UAE	Gallic acid, protocatechuic acid, *p*-coumaric acid, ferulic acid, quercetin, kaempferol, apigenin	(Saha et al., 2019)
*Olea europaea*	ChCl	Acetic acid	1:200	1:2 and 50 % (w/w)	180	54.1	MA	Oleopein, ferulic acid, kaempferol, oleuropein, luteolin, tyrosol	(de Almeida Pontes et al., 2021)
*Solanum tuberosum*	ChCl	Triethylene glycol	1:10	1:4 and 30 % (v/v)	30	40	UAE	Chlorogenic acid, cryptochlorogenic acid, *p*-coumaric acid, caftaric acid, neochlorogenic acid, caffeic acid, sinapic acid, ferulic acid, gallic acid, protocatechuic acid, gentisic acid, 4-hydroxybenzoic acid, vanillic acid, benzoic acid, vanillin, rutin, catechin, daidzein, quercetin, ellagic acid, myricetin, baicalein, apigenin, hesperidin, naringenin, isovitexin, resveratrol	(Suo et al., 2022)
*Moringa oleifera* leaves	L- Proline	Glycerol	1:10	2:5 and 37 % (*v*/v)	30	40	UAE	(−)-Epigallocatechin, gallic acid, D-(+)-catechin, vicenin-2, *p*-hydroxybenzoic acid, orientin, rutin, hyperoside, kaempferol-3-rutinoside, isorhamnetin-3-glucoside, rosmarinic acid, quercetin, apigenin, kaempferol	(Wu et al., 2020)
*Citrus limon*	ChCl	Glycerol	1:13	3:1 and 55 % (*w*/*v*)	36	50	MA	Apigenin, epicatechin, catechin, ferulic acid, *p*-coumaric acid, quercetin, diosmin, sinapic acid, gallocatechin, rutin, luteolin, quercitrin	(Kalogiouri et al., 2022)

As shown in Table 1, a previous investigation reported the flexibility of DES extraction, with small changes in the DES composition significantly changing the extracted polyphenols. Acid-based DES formulations, particularly ChCl-p-toluenesulfonic acid (1:2 M ratio), demonstrated enhanced flavonoid extraction efficiency, yielding myricetin (57.2 mg/g), morin (12.7 mg/g), and rutin (9.1 mg/g) from *Lycium barbarum* via ultrasound-assisted extraction at 25 °C for 90 min, with superior performance attributed to strong proton-donating capacity and optimized physicochemical properties (Ali et al., 2019). DES extraction exhibits remarkable flexibility, with small compositional changes significantly shifting extracted polyphenol profiles: ChCl:urea targets chlorogenic acids, ChCl:glucose targets phenolic acids, and ChCl:citric acid targets rutin, each via 30-min UAE at 25 °C (Ianni et al., 2023).

Extraction method selection critically influences DES performance. All DES formulations assisted by ultrasound-assisted extraction (UAE) or maceration yielded superior total phenolic content (TPC) compared to conventional methanol or ethanol extraction. From *Mangifera indica* seeds, ChCl:ethylene glycol via maceration achieved highest TFC and TPC yields; from fruit peel, β-alanine:malic acid:water combined with UAE yielded optimal results. However, high-viscosity formulations (e.g., β-alanine:citric acid:water) showed reduced efficiency with UAE due to decreased ultrasound-induced cavitation (Ojeda et al., 2023). Temperature and water content optimization are essential: UAE-DES extraction of *Aegle marmelos* via ChCl:oxalic acid achieved maximum TPC at 25 % (v/v) water and 80 °C, with elevated temperatures and water addition decreasing DES viscosity and increasing diffusion rates (Saha et al., 2019).

DES offer additional benefits including enhanced handling safety and versatility. Nevertheless, DES recovery presents significant technical challenges due to negligible vapor pressure and strong hydrogen bonding between HBA and HBD components, which preclude separation by conventional distillation. Membrane processes (ultrafiltration, nanofiltration) have emerged as promising approaches for DES recycling (Elizondo Sada et al., 2024).

Polyphenol recovery from DES can be performed via resin adsorption (Rodríguez-Juan et al., 2021; Wang et al., 2020a), liquid–liquid extraction with cosolvents (Pareja-Sánchez et al., 2025), or antisolvent precipitation (Ali et al., 2019). Liquid-liquid extraction requires substantial organic solvent consumption, rendering it environmentally unfavorable at industrial scale (Zhou et al., 2023). Macroporous resin (MPR) adsorption is the most efficient polyphenol recovery method, with easily recovered and reusable desorption solvents (Alam et al., 2021; Ali Redha, 2021; Della Posta et al., 2022). A sequential extraction strategy combining Soxhlet (methanol) with DES-UAE (lactic acid:glycerol:water) demonstrates DES’ anticipated replacement of traditional solvents in pharmaceutical, cosmetic, and food industries (Rebocho et al., 2022). Foodstuff-derived DES offer advantages through cost-effectiveness, nontoxicity, and potential to increase nutritional value of recovered compounds.

### 3.2 Subcritical water extraction

**Fig. 5:**
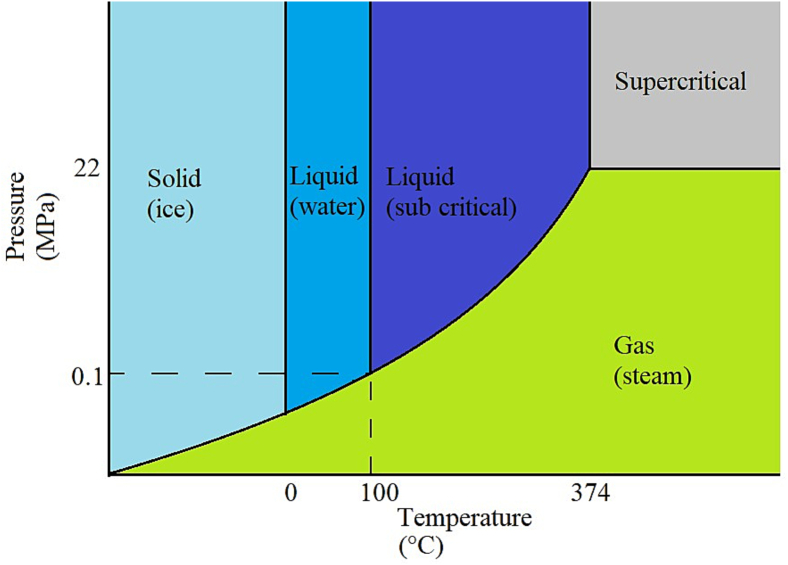
– Phase diagram for water at various pressures and temperatures. The dark blue area is the subcritical regime for water. Figure adapted from previous research Thiruvenkadam et al. (2015). (For interpretation of the references to color in this figure legend, the reader is referred to the web version of this article.)

Subcritical water extraction (SWE) represents a relatively novel and increasingly applied extraction method that leverages the altered physicochemical properties of water under elevated temperature and pressure conditions (100–374 °C and 0.1–22 MPa) while maintaining its liquid state (Žagar et al., 2024) (Fig. 5) By modulating temperature, pressure, viscosity, dielectric constant, surface tension, diffusivity, and pH, SWE enables water to mimic the solvent capabilities of conventional acids and organic solvents, thereby producing aqueous extracts free of hazardous organic solvent or residual acid contamination (Zhang et al., 2020). A critical advantage of SWE is its capacity to catalyze hydrolysis reactions, releasing ether- and ester-bound compounds from the lignocellulosic matrix. Water autoionization generates hydronium ions (H₃O^+^) that catalyze hemicellulose depolymerization and cleavage of acetyl groups, with liberated acetic acid further enhancing the acidic environment and facilitating lignin–hemicellulose bond cleavage (Hamzah et al., 2023; Sarker et al., 2021). This mechanism is particularly efficient in biomass with higher acetyl group content, enabling selective access to intracellular polyphenols without chemical additives or solvent regeneration (Sarker et al., 2021).

An overview of the relevant literature applying SWE to recover polyphenols from various foods and food wastes is presented in Table 2.

**Table 2:** – Optimal extraction parameters of polyphenols by subcritical water extraction. ND: No data.

**Botanical name**	**Temperature** **[°C]**	**Pressure** **[MPa]**	**Time** **[min]**	**Extracted polyphenols**	**Ref.**
*Punica granatum* seed residue	220	6	30	Caffeic acid derivatives, nicotiflorin	(He et al., 2012)
*Tagetes erecta* flower residue	220	ND	45	Quercetagetin, 6-hydroxykaempferol-3-glucoside, quercetin and patuletin-7-glucoside	(Xu et al., 2015)
*Olea europaea* pulp residue	160	ND	30	Chlorogenic acid, homovanillic acid, gallic acid, hydroxytyrosol, quercetin, syringic acid, oleuropein, genkwanin, caffeic acid	(Yu et al., 2015)
*Salicornia ramosissima*	140	2	30	Gallic acid, protocatechuic acid, neochlorogenic acid, catechin, caftaric acid, caffeine, chlorogenic acid, cryptochlorogenic acid, vanillic acid, caffeic acid, syringic acid, epicatechin, *p*-coumaric acid, ferulic acid, sinapic acid, polydatin, naringin, 3,5-di-caffeoylquinic acid, hyperoside, resveratrol, isoquercitrin, rutin, phloridzin, ellagic acid, 3,4-di-caffeoylquinic acid, myricetin, cinnamic acid, quercitrin, astragalin, isorhamnetin-3-glucoside, nicotiflorin, narcissin, naringenin, ε-viniferin, quercetin, phloretin, tiliroside, kaempferol, apigenin, chrysin	(Correia et al., 2022)
*Thymus vulgaris* byproduct	100	10	5	3,4-dihydroxyphenyllactic acid, linocaffein, caffeic acid, dihydrocaffeic acid, vicenin 2, isoquercitrin, eriocitrin, dihydrokaempferol-3-glucoside, cyranoside, luteolin-7-glucuronide, apigenin-7-glucuronide, rosmarinic acid, luteolin, cirsimaritin, fastigenin, pebrelin, carnosol	(Vergara-Salinas et al., 2012)
*Matricaria chamomilla*	150	4.5	30	Apigetrin, apigenin, astragalin, kaempferol, cyranoside, luteolin, naringin, naringenin, rutin, hyperoside, catechin, galangin, phloretin, resveratrol, aesculin, ellagic acid, *p*-hydroxybenzoic acid, protocatechuic acid, caffeic acid, sinapic acid, cinnamic acid, neochlorogenic acid, *p*-coumaric acid, coniferyl aldehyde	(Cvetanović et al., 2019)
*Zingiber officinale* byproduct	220	2	15	6-gingerol, 6-shogaol, 8-gingerol	(Sulejmanović et al., 2024)
*Fagopyrum tataricum*	220	5	60	Sinapic acid, epicatechin, gallic acid, syringic acid, caftaric acid, *p*-coumaric acid, astragalin, kaempferol, rutin, quercetin, isoorientin, orientin, hyperoside	(Dzah et al., 2020)
*Nelumbo nucifera*	120	ND	15	Procyanidin, catechin, kuromanin, isoquercitrin, astragalin, isorhamnetin	(Yan et al., 2020)

SWE demonstrates superior bioactivity profiles compared to conventional extraction methods. Multiple studies report 4-fold higher antioxidant activity and 2–3 fold higher polyphenol yields with significantly enhanced antiproliferative activity against HepG2 cells compared to hot water and ethanol extracts (Benito-Román et al., 2020;Yan et al., 2020; Žagar et al., 2024). Superior antimicrobial activity further positions SWE as advantageous for multi-functional extract production. Temperature-dependent selectivity enables extraction of structurally distinct polyphenols, exemplified by *Matricaria chamomilla* optimization at 150 °C for maximum antioxidant yield, with individual compounds demonstrating differential thermal stability: apigenin, luteolin, and naringin glucosides degrade above 85 °C, whereas corresponding aglycones (apigenin, luteolin, naringenin, kaempferol) degrade above 115°. Polyphenols such as phloretin and resveratrol demonstrate enhanced solubility in subcritical water at 210 °C due to specific molecular structure optimization (Cvetanović et al., 2019).

Optimization of SWE parameters – extraction temperature, residence time, and solid-to-solvent loading ratio - via Response Surface Methodology reveals that extraction time represents the most significant variable affecting polyphenol yield, followed by temperature and solid loading (Ren et al., 2024). Optimal extraction conditions are reported as approximately 160–220 °C for 16–45 min at 2–6 MPa, or alternatively 100–140 °C for 5–130 min at 10–15 MPa depending on pressure application (Batista et al., 2019). Elevated temperatures enhance polyphenol recovery through reduced water viscosity and increased diffusivity; however, temperatures exceeding optimal ranges induce selective thermal degradation of thermally unstable polyphenols and formation of neoformed thermal byproducts with unknown toxicological profiles (Yan et al., 2020; Žagar et al., 2024). This thermal sensitivity necessitates careful optimization balancing maximum polyphenol extraction against compound degradation minimization.

A critical limitation of SWE is the inherent thermal instability of heat-labile polyphenol classes, particularly flavonols and stilbenes, which undergo selective degradation at elevated temperatures and extended extraction times (Benito-Román et al., 2020). Additionally, conditions promoting biomass hydrolysis can paradoxically induce undesirable reactions leading to toxic byproduct formation or target compound degradation (Batista et al., 2019). The requirement for high-pressure vessels and specialized equipment creates substantial capital and operational costs, potentially limiting accessibility for small-scale applications. Despite these constraints, SWE stands out among lignocellulosic pretreatment methods as an environmentally superior option producing no chemical residues and requiring no solvent regeneration, positioning it as a sustainable alternative to conventional organic solvent-based extraction for industrial-scale polyphenol recovery.

### 3.3 Isolation by macroporous resin adsorption

Isolation by MPR adsorption represents a well-established purification methodology based on noncovalent bonding interactions – including hydrophobic interactions, π–π stacking, van der Waals forces, and hydrogen bonding - between polyphenols and the resin matrix. This technique enables selective concentration and recovery of bioactive polyphenolic compounds, facilitating their identification, characterization, and formulation as natural drug substances or nutraceutical ingredients with enhanced efficacy per unit mass compared to crude preparations (Aljawarneh et al., 2024). The adsorption process occurs through sequential mass transfer steps: i) adsorbate transport from solution to the MPR boundary layer, ii) mass transport through the boundary layer, iii) adsorbate diffusion into pores, and iv) adsorption and desorption of adsorbate (Soto et al., 2011). Adsorption kinetics are highly dependent on the adsorbent's molecular matrix and specific affinity mechanisms, with mass transfer controlled by external diffusion through the liquid film and intraparticle pore diffusion, both significantly influenced by fluid dynamics and resin microstructure (Jakovljević Kovač et al., 2021).

Planar noncovalent π–π interactions form between benzene rings at typical distances of 3.00–3.46 Å, with π-electron clouds aligning in parallel and creating attractive forces between electron-rich and electron-deficient regions. Amberlite XAD macroporous resins - composed of styrene–divinylbenzene, aliphatic esters, phenol−formaldehyde, or acrylic ester matrices -are highly cross-linked and nonpolar (with two slightly polar exceptions), enabling robust π–π affinity bonding with polyphenolic compounds. Resin selection critically influences adsorption efficiency; moderate polar resins with acrylic matrices (e.g., XAD7HP) exhibit stronger affinity for flavonoids than non-polar polystyrene-based XAD4, as polarity matching governs sorption via hydrophobic and polar interactions (Che Zain et al., 2020). XAD-2, XAD-4, XAD-16, and XAD-1180 are all capable of forming π–π affinity bonds with polyphenols, as their MPR matrices are composed of styrene–divinylbenzene, which constitutes the nonpolar backbone of these resins (see Table 3).

**Table 3:** – Different Amberlite XAD MPRs and their properties. NA: Not applicable.

MPR	Matrix	Surface area (m^2^/g)	Pore diameter (Å)	Target molecular weight (MW)	Ref.
XAD-2	Styrene–divinylbenzene	330	90	<20.000	(Sigma-Aldrich Corp, 2025; Tungmunnithum et al., 2020; Wang et al., 2024)
XAD-4	Styrene–divinylbenzene	725	90	NA	(Santos et al., 2022; Seif Zadeh & Zeppa, 2022; Sigma-Aldrich Corp; Tungmunnithum et al., 2020; Wang et al., 2024; Xu et al., 2019)
XAD-7	Aliphatic ester	450	50	<60.000	(Della Posta et al., 2022; Gao et al., 2018; Li & Chase, 2010; Santos et al., 2022; Seif Zadeh & Zeppa, 2022; Sigma-Aldrich Corp; Tang et al., 2012; Tungmunnithum et al., 2020; Wang et al., 2024; Xu et al., 2019)
XAD-16	Styrene–divinylbenzene/ Polystyrene	900	100	<40.000	(Della Posta et al., 2022; Gao et al., 2018; Li & Chase, 2010; Sandhu & Gu, 2013; Santos et al., 2022; Seif Zadeh & Zeppa, 2022; Sigma-Aldrich Corp; Tang et al., 2012; Tungmunnithum et al., 2020; Wang et al., 2024)
XAD-761	Phenol− formaldehyde	200	600	NA	(Gao et al., 2018; Sandhu & Gu, 2013; Santos et al., 2022)
XAD-1180	Styrene–divinylbenzene	600	300	NA	(dos Santos et al., 2022; Gao et al., 2018; Li & Chase, 2010; Sigma-Aldrich Corp, 2025; Tang et al., 2012; Wang et al., 2024)

During adsorption, larger molecules and high molecular weight components present in crude extracts—such as proteins and lignocellulosic material—are physically excluded from resin pores based on size. Many polar compounds pass through without interaction. In contrast, flavonoids with multiple aromatic rings form π–π conjugation interactions with styrene–divinylbenzene matrices, while simultaneously establishing van der Waals forces and hydrogen bonding with acrylic ester-based resins, enabling selective adsorption and high enrichment (Che Zain et al., 2020). Dynamic adsorption-desorption protocols using volumetric flow rate optimization (e.g., 1.5 BV/h for XAD-16 desorption) have been established, demonstrating scalability from batch to pilot and industrial-scale applications via column chromatography (Seif Zadeh & Zeppa, 2022). Importantly, organic solvents used for polyphenol desorption can be easily recovered and reused, reducing operational costs and environmental impact (Alam et al., 2021).

Table 4 shows an overview of the extraction and adsorption studies of different XAD MPRs.

**Table 4:** – Adsorption isotherm experiments. Overview of MPR Amberlite XADs featuring studies where polyphenols are adsorbed. DM: dry matter. TFC: Total flavonoid content; TPC: Total polyphenol content; TAC: Total anthocyanin content; NADES: Natural deep eutectic solvents; UAE: Ultrasound-assisted extraction; TPCC: Total procyanidin content.

**Botanical name**	**Extraction method**	**Polyphenols**	**Adsorption efficiency**	**Ref.**
XAD-2				
*Nymphaea lotus*	100 mg DM in 1 mL 90 % (*v*/v) EtOH, 45 °C at 30 kHz UAE for 46 min	TFC, isorhamnetin-3-galactoside, isorhamnetin-3-xyloside, myricetin-3-galactoside, myricetin-3-xyloside, quercetin-3-xyloside, quercitrin, trifolin	Best candidate. Resembled XAD-7.	(Tungmunnithum et al., 2020)
*Triticum aestivum* straw	3.5 g DM mixed with 70 g of the ionic liquid 1-ethyl-3-methylimidazolium acetate, 120 °C for 6 h	Tricin, vanillin, p-hydroxybenzaldehyde, syringaldehyde	Was outperformed by XAD-7	(da Costa Lopes et al., 2016)
*Apis mellifera* honey from *Brassica napus*	Dissolved in water, 60 g to 600 mL solution	TFC	Best candidate of six MPR	(Lv et al., 2018)
XAD-4				
*Nymphaea lotus*	100 mg DM in 1 mL 90 % (*v*/v) EtOH, 45 °C at 30 kHz UAE for 46 min	TFC, isorhamnetin-3-galactoside, isorhamnetin-3-xyloside, myricetin-3-galactoside, myricetin-3-xyloside, quercetin-3-xyloside, quercitrin, trifolin	Was slightly outperformed by XAD-7 and XAD-2. Resembled XAD-16	(Tungmunnithum et al., 2020)
*Eucalyptus globulus*	52 % (v/v) EtOH extract by ultrafiltration in diafiltration mode	TPC	Was outperformed by XAD-16 N	(Pinto et al., 2017)
*Glycyrrhiza glabra* leaf	100 g DM in 2 L 80 % (v/v) EtOH, 60 °C, 800 W UAE, 90 min	TFC, pinocembrin	Resembled XAD-16	(Dong et al., 2015)
*Olea europaea* mill wastewater	Lipids were removed, and the wastewater was acidified to pH 3, followed by sedimentation by gravity	Lutolin, cynaroside, caffeic acid, vanillic acid, tyrosol, hydroxytyrosol, oleuropein	Resembled XAD-16 and XAD-7, and was outperformed XAD-761	(Kaleh & Geißen, 2016)
*Solanum tuberosum* tubers	80 % EtOH, maceration, 1:5 *w*/w solid:solvent, 24 h	TAC, petunidin-3-(p-coumaroyl)-rutinoside-5-glucoside, peonidin-3-(p-coumaroyl)-rutinoside-5-glucoside	Outperformed by XAD-7 and XAD-16	(Heinonen et al., 2016)
*Liriodendron tulipifera*	The biomass was milled to 20–80 mesh and deacetylated at 60 °C in 0.8 w% NaOH in 1:8 w/w solid:liquid, followed by hydrothermal pretreatment at 170 °C for 60 min in 1:8 solid:water.	TPC, isovanillic acid, 3,5-dimethoxyphenol, 2-methyl-4-(1,1,3,3-tetramethylbutyl)- phenol, 3,5-dimethoxy-4-[(trimethylsilyl)oxy]-benzaldehyde, syringic acid-di-trimethylsilyl	Outperformed by XAD-16	(Um et al., 2017)
*Citrus × sinensis* fruit juice	No extraction	Naringenin	Outperformed by XAD-7	(Ribeiro et al., 2002)
*Citrus × sinensis* and *Citrus x paradisi* peel, juice, and molasses	Diluted to 25–30°Brix using water and centrifuged at 14000 *g* for 20 min	p-Hydroxycinnamic acid derivatives and flavonoids by ion exchange chromatography	Outperformed by XAD-7 and XAD-16	(Grohmann et al., 1999)
*Beta vulgaris* var. Lutea	Dark maceration with water added hydrochloric acid to pH 3.5, 1:1–1:1.5 w/w solid:solvent, 12 h, filtered and centrifuged at 3500 rpm for 30 min	Betaxanthin	Outperformed by XAD-16	(Li et al., 2021)
*Salicornia ramosissima*	800 g biomass extracted by one percolation of 20 L by dynamic subcritical water extraction at 140 °C, 30 min cycle time, at 17 bar	Protocatechuic acid, p-coumaric acid, vanillic acid, caffeic acid, ferulic acid, quercetin, isorhamnetin, neochlorogenic acid, cryptochlorogenic acid, chlorogenic acid, astragalin, hyperoside, isoquercitrin	Only candidate	(Fredsgaard, Tchoumtchoua, et al., 2023)
XAD-7				
*Inga edulis* leaves	Dark maceration 5 g in 120 mL 50 % (*v*/v) EtOH, 50 °C for 2 h	TFC, TPC	Best candidate	(Silva et al., 2007)
*Nymphaea lotus*	100 mg DM in 1 mL 90 % (*v*/v) EtOH, 45 °C at 30 kHz UAE for 46 min	TFC, isorhamnetin-3-galactoside, isorhamnetin-3-xyloside, myricetin-3-galactoside, myricetin-3-xyloside, quercetin-3-xyloside, quercitrin, trifolin	Best candidate. Resembled XAD-2.	(Tungmunnithum et al., 2020)
*Triticum aestivum* straw	3.5 g DM mixed with 70 g of the ionic liquid 1-ethyl-3-methylimidazolium acetate, 120 °C for 6 h	Tricin, vanillin, p-hydroxybenzaldehyde, syringaldehyde	Outperformed by XAD-2	(Pérez-Larrán et al., 2018)
*Solanum tuberosum* tubers	80 % EtOH, maceration, 1:5 w/w solid to solvent, 24 h	TAC, petunidin-3-(p-coumaroyl)-rutinoside-5-glucoside, peonidin-3-(p-coumaroyl)-rutinoside-5-glucoside	Best candidate	(Heinonen et al., 2016)
*Olea europaea* mill wastewater	Lipids were removed, and the wastewater was acidified to pH 3, followed by sedimentation by gravity	Luteolin, luteolin-7-glucoside, caffeic acid, vanillic acid, tyrosol, hydroxytyrosol, oleuropein	Resembled XAD-4 and XAD-16. Outperformed XAD-761	(Kaleh & Geißen, 2016)
*Citrus × sinensis* fruit juice	No extraction	Naringenin	Best candidate	(Ribeiro et al., 2002)
*Citrus × sinensis* and *Citrus x paradisi* peel, juice, and molasses	Diluted to 25–30°Brix using water and centrifuged at 14000 g for 20 min	p-Hydroxycinnamic acid derivatives and flavonoids by ion exchange chromatography	Outperformed by XAD-16	(Grohmann et al., 1999)
*Spinacia oleracea*	Maceration with water added phosphoric acid to pH 2, 2 kg DM to 2 L acidified water, 70 °C, 1 h	Patuletin, spinacetin	Resembled XAD-16	(Aehle et al., 2004)
*Brassica oleracea* var. *capitata* f. *rubra*	Juiced in a home juicer and centrifuged at 5000 rpm for 20 min, the supernatant was acidified to pH 3	TAC	Best candidate	(Coutinho et al., 2004)
*Citrus unshiu* fruit peels	30 g DM in 300 mL water, maceration at 62 °C for 30 min	Narirutin, hesperidin	Best candidate	(Kim et al., 2007)
*Morus nigra* berry	10 g biomass in 1 L 0.1 w% HCl 70 % (*v*/v) EtOH, maceration, room temperature, 24 h	TAC, kuromanin, keracyanin, cyanidin	Best candidate of five MPRs	(Chen et al., 2016)
*Myrciaria cauliflora* fruit by-product	Solid-to-solvent ratio of 1:15:15 (*w*/*v*/v), NADES solvent being choline chloride and propylene glycol (1:1) and water, maceration, 50 °C for 60 min	Kuromanin	Only candidate	(Benvenutti et al., 2020)
*Vitis rotundifolia* juice pomace	200 g biomass in 500 mL 1 w% CH_2_O_2_, UAE at 90 °C twice for 15 min	TAC, TPC, delphinidin-3,5-diglucoside, cyanin, pelargonin, petunin, peonin, malvin, ellagic acid, myricetin, quercetin, kaempferol, catechin, epicatechin	Was outperformed by XAD 16, XAD-761, XAD-1180	(Sandhu & Gu, 2013)
*Vaccinium* subgenus *oxycoccus* pomace	400 g biomass in 1 L 1 w% CH_2_O_2_, UAE at 90 °C twice for 10 min	TPC, TPCC, peonidin-3-glucoside, myrtillin, kuromanin, callistephin, petunidin-3-glucoside, malvin, kaempferol, quercetin, myricetin, ellagic acid, caffeic acid, resveratrol	Best candidate	(Gao et al., 2018)
*Rubus ursinus × R. idaeus* seeds and juice	Maceration, 60 % EtOH	Peduculagin, peduculagin isomer, sanguiin H-6, sanguiin H-10, lambertianin A, bis-HHDP-glucose, sanguiin H.2, lambertianin C	Only MPR used	(Furuuchi et al., 2011)
Oak aged *Vitis vinifera* wine	Fermentation	Vescalagin, castalagin, acutissimin, epiacutissimin A,epiacutissimin B, ethylvescalagin	Only MPR used	(Saucier et al., 2006)
XAD-761				
*Olea europaea* mill wastewater	Lipids were removed, and the wastewater was acidified to pH 3, followed by sedimentation by gravity	Lutolin, cynaroside, caffeic acid, vanillic acid, tyrosol, hydroxytyrosol, oleuropein	Was outperformed by XAD-4, XAD-16 and XAD-7	(Kaleh & Geißen, 2016)
*Vitis rotundifolia* juice pomace	200 g biomass in 500 mL 1 w% CH_2_O_2_, UAE at 90 °C twice for 15 min	TAC, TPC, delphinidin-3,5-diglucoside, cyanin, pelargonin, petunin, peonin, malvin, ellagic acid, myricetin, quercetin, kaempferol, catechin, epicatechin	Better than XAD-7, same as XAD-1180, outperformed by XAD-16 N	(Sandhu & Gu, 2013)
*Vaccinium* subgenus *oxycoccus* pomace	400 g biomass in 1 L 1 w% CH_2_O_2_, UAE at 90 °C twice for 10 min	TPC, TPCC, peonidin-3-glucoside, myrtillin, kuromanin, callistephin, petunidin-3-glucoside, malvin, kaempferol, quercetin, myricetin, ellagic acid, caffeic acid, resveratrol	Outperformed by XAD-16 N, XAD-761, XAD-1180	(Gao et al., 2018)
XAD-16				
*Inga edulis* leaves	Dark maceration 5 g in 120 mL 50 % (v/v) EtOH, 50 °C for 2 h	TFC, TPC	Was outperformed by XAD-7	(Silva et al., 2007)
*Nymphaea lotus*	100 mg DM in 1 mL 90 % (v/v) EtOH, 45 °C at 30 kHz UAE for 46 min	TFC, isorhamnetin-3-galactoside, isorhamnetin-3-xyloside, myricetin-3-galactoside, myricetin-3-xyloside, quercetin-3-xyloside, quercitrin, trifolin	Was slightly outperformed by XAD7 and XAD-2. Resembled XAD-4.	(Tungmunnithum et al., 2020)
*Eucalyptus globulus*	52 % (v/v) EtOH extract by ultrafiltration in diafiltration mode	TPC	Best candidate	(Pinto et al., 2017)
*Glycyrrhiza glabra* leaf	100 g DM in 2 L 80 % (v/v) EtOH, 60 °C, 800 W UAE, 90 min	TFC, pinocembrin	Resembled XAD-4	(Dong et al., 2015)
*Mangifera indica* fruit peel	125 mg DM in 8 mL 80 % (v/v) MeOH, UAE for 3 times of 15 s, each time introducing fresh solvent	Gallic acid, digallic acid, methyl gallate, monogalloyl glucose, Penta-galloyl glucose, mangiferin	Outperformed by five other Lewatit® MPRs	(Geerkens et al., 2015)
*Olea europaea* mill wastewater	Lipids were removed, and the wastewater was acidified to pH 3, followed by sedimentation by gravity	Lutolin, cynaroside, caffeic acid, vanillic acid, tyrosol, hydroxytyrosol, oleuropein	Resembled XAD-4 and XAD-7. Outperformed XAD-761	(Kaleh & Geißen, 2016)
*Solanum tuberosum* tubers	80 % EtOH, maceration, 1:5 *w*/w solid to solvent, 24 h	TAC, petunidin-3-(p-coumaroyl)-rutinoside-5-glucoside, peonidin-3-(p-coumaroyl)-rutinoside-5-glucoside	Best candidate	(Heinonen et al., 2016)
*Liriodendron tulipifera*	The biomass was milled to 20–80 mesh and deacetylated at 60 °C in 0.8 w% NaOH in 1:8 w/w solid:liquid, followed by hydrothermal pretreatment at 170 °C for 60 min in 1:8 solid:water.	TPC, isovanillic acid, 3,5-dimethoxyphenol, 2-methyl-4-(1,1,3,3-tetramethylbutyl)-phenol, 3,5-dimethoxy-4-[(trimethylsilyl)oxy]-benzaldehyde, syringic acid-di-trimethylsilyl	Best candidate	(Um et al., 2017)
*Beta vulgaris* var. Lutea	Dark maceration with water added hydrochloric acid to pH 3.5, 1:1–1:1.5 w/w solid:solvent, 12 h, filtered and centrifuged at 3500 rpm for 30 min	Betaxanthin	Best candidate	(Li et al., 2021)
*Citrus × sinensis* fruit juice	No extraction	Naringenin	Outperformed by XAD-7	(Ribeiro et al., 2002)
*Citrus × sinensis* and *Citrus x paradisi* peel, juice, and molasses	Diluted to 25–30°Brix using water and centrifuged at 14000 g for 20 min	p-Hydroxycinnamic acid derivatives	Best candidate	(Grohmann et al., 1999)
*Malus domestica* fruit pomace	20 g DM in 70 % (v/v) acetone, maceration, 60 min	Quercetin, hyperoside, quercitrin, isoquercitrin, rutin, quercetin-3-xyloside, avicularine, protocatechuic acid, p-hydroxybenzoic acid, caffeic acid, p-coumaric acid, ferulic acid, phloretin, catechin, epicatechin, chlorogenic acid, procyanidin B1, procyanidin B2, phloridzin	Only candidate	(Schieber et al., 2003)
*Spinacia oleracea*	Maceration with water added phosphoric acid to pH 2, 2 kg DM to 2 L acidified water, 70 °C, 1 h	Patuletin, spinacetin	Resembled XAD-7	(Aehle et al., 2004)
*Olea europaea* oil	Solid-to-solvent ratio of 1:1 (w/v), NADES solvent being choline chloride, xylitol, and water (2:1:3 w/*w*/w), maceration, 40 °C for 60 min	Hydroxytyrosol, tyrosol, oleacein, oleocanthal, luteolin, apigenin, pinoresinol, 1-acetoxypinoresinol	Only candidate	(Rodríguez-Juan et al., 2021)
*Camellia oleifera* seed oil	Solid-to-solvent ratio of 1:1 (*w*/*v*), NADES solvent being choline chloride and glycerol (1:2 w/w), maceration, 50 °C for 60 min	Gallic acid, p-hydroxybenzoic acid, protocatechuic acid, benzoic acid, phthalic acid, *p*-hydroxyphenylacetic acid, vanillic acid, cinnamic acid, p-coumaric acid, caffeic acid, ferulic acid, sinapic acid, chlorogenic acid, apigenin, kaempferol, naringenin, luteolin, quercetin, myricetin, vitexin, taxifolin, catechin, epicatechin, epigallocatechin gallate, epigallocatechin	Only candidate	(Wang et al., 2020a)
*Vitis rotundifolia* juice pomace	200 g biomass in 500 mL 1 w% CH_2_O_2_, UAE at 90 °C twice for 15 min	TAC, TPC, delphinidin-3,5-diglucoside, cyanin, pelargonin, petunin, peonin, malvin, ellagic acid, myricetin, quercetin, kaempferol, catechin, epicatechin	Best candidate	(Sandhu & Gu, 2013)
*Vaccinium* subgenus *oxycoccus* pomace	400 g biomass in 1 L 1 w% CH_2_O_2_, UAE at 90 °C twice for 10 min	TPC, TPCC, peonidin-3-glucoside, myrtillin, kuromanin, callistephin, petunidin-3-glucoside, malvin, kaempferol, quercetin, myricetin, ellagic acid, caffeic acid, resveratrol	Outperformed by XAD-7, better than XAD-761, XAD-1180	(Gao et al., 2018)
XAD-1180				
*Vitis rotundifolia* juice pomace	200 g biomass in 500 mL 1 w% CH_2_O_2_, UAE at 90 °C twice for 15 min	TAC, TPC, delphinidin-3,5-diglucoside, cyanin, pelargonin, petunin, peonin, malvin, ellagic acid, myricetin, quercetin, kaempferol, catechin, epicatechin	Same as XAD-761, better than XAD-7, outperformed by XAD-16	(Sandhu & Gu, 2013)
*Vaccinium* subgenus *oxycoccus* pomace	400 g biomass in 1 L 1 w% CH_2_O_2_, UAE at 90 °C twice for 10 min	TPC, TPCC, peonidin-3-glucoside, myrtillin, kuromanin, callistephin, petunidin-3-glucoside, malvin, kaempferol, quercetin, myricetin, ellagic acid, caffeic acid, resveratrol	Outperformed by XAD-7, XAD 16 N better than XAD-761	(Gao et al., 2018)

As seen from Table 4, owing to the complexity of the individual systems, it can be challenging to estimate the most suitable MPR Amberlite XAD for a given extract solely on the basis of the available literature. Therefore, screening individual extracts against several XAD MPRs of interest is necessary to identify the optimal candidate. The analysis of 51 documented polyphenol isolations reveals that XAD-7 and XAD-16 demonstrate superior overall performance: XAD-7 was optimal for 16 isolations, while XAD-16 was optimal for 17, reflecting their dominance in polyphenol adsorption capacity. According to Table 3 and Table 4, XAD-16 has a larger surface area and pore size than XAD-2, XAD-4, and XAD-7. The larger surface area and pore diameter of XAD-16 favor π–π interactions with polyphenolic benzene rings and improve fluid dynamics within sorption processes, enhancing absorption rates and capacities. XAD-7 has a slightly polar surface, which favors strong hydrogen bonds with the hydroxyl groups of polyphenols (DuPont, 2025; Seif Zadeh & Zeppa, 2022).

Isotherm models can be fitted to the obtained adsorption data for spectrophotometric assays. Furthermore, individual polyphenols can be qualitatively and quantitatively determined via methods such as LC–MS, which allows for the determination of molecule-specific isotherms. In Table 5, isotherms of XAD-7 and XAD-16 are shown for different extracts.

**Table 5:** – Overview of literature data for dynamic adsorption experiments using Amberlite XAD MRPs including volumetric flow rates and 5 % breakpoint points.

MPR	Botanical name	Polyphenol	Flowrate (BV/h)	Breakpoint cut-off (BV)	Ref.
XAD-7	*Vaccinium* subgenus *oxycoccus* pomace	Procyanidins	0.25 0.35 0.45	28 21 15	(Gao et al., 2018)
XAD 16	*Beta vulgaris* var. Lutea	Betaxanthin	1 2	12.6 5.0	(Li et al., 2021)
	Roasted nut *Corylus avellana* skin	Total polyphenol content	1.5 3 5	3.0 3.8 3.8	(Seif Zadeh & Zeppa, 2022)

In all the studies listed in Table 5, the volumetric flow rates were varied to investigate their effects. In one out of the four studies, pure compounds, identified by LC–MS, were utilized to calculate breakpoint cutoffs and breakthrough curves. An initial screening of suitable XAD MPRs via adsorption isotherm experiments was conducted prior to the dynamic adsorption experiments. In the experiment where XAD-16 was tested for the adsorption of betaxanthin, the 5 % breakpoint cutoff decreased with increasing volumetric flow rates, indicating that adsorption was highly sensitive to changes in the flow rate (Li et al., 2021). XAD-7 demonstrated, in two cases, the ability to absorb flavonoid aglycones and procyanidins (Gao et al., 2018; Li & Chase, 2009). Both compound types contain at least two hydroxyl groups, enabling them to form noncovalent intermolecular bonds with the XAD-7 surface.

A significant limitation of MPR purification is the inherent trade-off between selectivity and chemical diversity. Selective isolation achieves high enrichment of target polyphenols with concentrated bioactivity per unit mass; however, this selectivity results in loss of *co*-extracted minor polyphenols, potentially eliminating polyvalent bioactivity profiles and synergistic interactions between compounds that characterize the original plant extract (Aljawarneh et al., 2024). Due to the complexity and variability of individual extract systems, screening individual crude extracts against multiple XAD MPRs is necessary to identify the optimal candidate, as no single resin universally performs optimally across diverse polyphenol matrices. Additionally, variations in surface area and pore diameter (≈10 % across literature) may influence adsorption performance (Batista et al., 2019; Della Posta et al., 2022; Meng et al., 2021; Sandhu & Gu, 2013; dos Santos et al., 2022; Seif Zadeh & Zeppa, 2022; Sigma-Aldrich Corp; Tang et al., 2012; Wang et al., 2024; Xu et al., 2019). Nevertheless, MPR adsorption remains the most cost-effective and operationally straightforward purification method for generating botanical drug substances and nutraceutical ingredients with standardized polyphenol content and enhanced bioactivity, bridging the gap between crude extract production and pharmaceutical formulation.

### 3.4 Comparison of extraction and isolation methods

Table 6 presents a comparative analysis of three advanced polyphenol extraction and isolation methodologies, DES, SWE, and MPR, evaluated across key performance parameters including typical yields, advantages, scalability, environmental impact, and limitations. DES excels in selectivity and generation of multi-functional extracts with dual antioxidant and antimicrobial activities, making them particularly advantageous for food waste valorization where extraction of diverse polyphenol classes enhances end-product utility. However, DES requires careful viscosity management - typically through controlled water addition or elevated temperatures - and solvent recovery optimization via emerging membrane technologies, which increases operational complexity and costs for industrial food waste processing. SWE offers eco-friendly extraction from food and food waste biomass with superior bioactivity profiles and no chemical residues, positioning it as an environmentally sustainable alternative for large-scale industrial applications. Nevertheless, SWE requires temperature-dependent optimization to balance maximum polyphenol recovery from food waste matrices against thermal degradation of heat-labile compounds, demanding precise parameter control for each specific biomass type. MPR provides cost-effective purification and concentration of polyphenols recovered from food waste extracts, with reusable matrices and easily recoverable desorption solvents reducing operational costs. However, MPR's selectivity-driven isolation sacrifices synergistic compound interactions by eliminating minor polyphenols from crude food waste extracts, potentially reducing the holistic bioactivity benefits that characterize multi-component natural extracts.

**Table 6:** - Comparative analysis of three advanced polyphenol extraction and isolation methods: DES, SWE, and MPR.

**Method**	**Exemplary Yields**	**Advantages**	**Scalability**	**Environmental Impact**	**Key Limitations**	**References**
**Deep Eutectic Solvents (DES)**	Myricetin: 57.2 mg/g, Morin: 12.7 mg/g, Rutin: 9.1 mg/g (*Lycium barbarum*, ChCl:p-toluenesulfonic acid 1:2, UAE 90 min, 25 °C)	High selectivity for polyphenols of diverse polarities via H-bonding; superior performance vs. conventional organic solvents ; dual functionality (antioxidant + antimicrobial: 10-fold higher activity vs. ethanol/water against *Bacillus subtilis* and *B. cereus*) ; enhanced handling safety; small compositional changes enable targeted extraction (chlorogenic acids, phenolic acids, rutin) ; flexibility for multi-functional food ingredients	DES extraction commonly assisted by UAE, MAE, maceration; Soxhlet+DES sequential strategy demonstrates industrial viability ; cost-effective, nontoxic foodstuff-derived DES (sugars, organic acids, amino acids, polyols)	Biodegradable, non-toxic components; negligible organic solvent use; potential to replace traditional solvents	High viscosity (>0.1 Pa·s) hinders diffusion and mass transfer ; adding >50 % *v*/v water disrupts polarity and H-bonds ; DES recovery via conventional distillation not feasible due to negligible vapor pressure ; liquid-liquid extraction requires substantial organic solvent consumption (environmentally unfavorable) ; membrane processes (ultrafiltration, nanofiltration) emerging as alternatives	(Ali et al., 2019; Coscarella et al., 2023; de Almeida Pontes et al., 2021; Elizondo Sada et al., 2024; García-Roldán et al., 2022; Ianni et al., 2023; Islamčević Razboršek et al., 2020; Ling & Hadinoto, 2022; Rebocho et al., 2022; Zhou et al., 2023)
**Subcritical Water Extraction (SWE)**	Optimal conditions: 160–220 °C, 16–45 min, 2–6 MPa; or 100–140 °C, 5–130 min, 10–15 MPa depending on pressure ; 4-fold higher antioxidant activity and 2–3 fold higher polyphenol yields vs. hot water and ethanol ; 150 °C optimal for *Matricaria chamomilla* antioxidant yield	Eco-friendly option producing no residues; no solvent regeneration required unlike other hydrolysis methods ; water autoionization generates H₃O^+^ catalyzing hydrolysis reactions ; enhanced antiproliferative activity against HepG2 cells vs. hot water and ethanol ; superior antimicrobial activity ; hemicellulose depolymerization via hydrolysis releases bound polyphenols ; selective biomass hydrolysis accessing intracellular polyphenolstemperature-dependent selectivity enables extraction of structurally distinct polyphenols (e.g., aglycones at 210 °C)	Scalable technology with RSM optimization for multiple parameters (temperature, residence time, solid-to-solvent loading) ; industrial applicability demonstrated	Water-based, environmentally friendly; no hazardous organic solvents; produces aqueous extract free of solvent/acid residues ; mimics solvent capabilities of acids and organic solvents	Elevated temperatures and pressures induce selective thermal degradation of heat-labile polyphenols (flavonols, stilbenes) ; risk of neoformed thermal byproducts with unknown toxicological profiles ; individual phenolic compounds show differential thermal stability (apigenin, luteolin, naringin glucosides degrade >85 °C; aglycones >115 °C) ; conditions leading to biomass hydrolysis can induce undesirable reactions and target compound degradation ; high capital and operating costs for pressure vessels and equipment[implied]	(Batista et al., 2019; Benito-Román et al., 2020; Cvetanović et al., 2019; Hamzah et al., 2023; Ren et al., 2024; Sarker et al., 2021; Yan et al., 2020; Žagar et al., 2024; Zhang et al., 2020)
**Macroporous Resin Adsorption (MPR)**	High enrichment of target polyphenols with concentrated bioactivity per unit mass; XAD-7 optimal for 16 out of 51 isolations reviewed; XAD-16 optimal for 17 out of 51 isolations ; mass transfer kinetics controlled by external diffusion and intraparticle pore diffusion	Cost-effective and operationally simple ; selective concentration and recovery via noncovalent bonding (hydrophobic, π–π stacking, van der Waals, H-bonding) ; high enrichment enabling formulation as botanical drug substances or nutraceutical ingredients ; XAD-7 and XAD-16 demonstrate superior performance across diverse polyphenol classes ; larger surface area and pore size of XAD-16 improves fluid dynamics and absorption rates ; XAD-7 slightly polar surface enables strong H-bonds with hydroxyl groups	Batch and dynamic adsorption protocols established; volumetric flow rate optimization feasible (e.g., 1.5 BV/h for XAD-16 desorption) ; scalable to pilot and industrial scales via column chromatography	Reusable resin matrices enable multiple adsorption-desorption cycles; organic solvents used for desorption can be easily recovered and reused ; eliminates need for further polyphenol purification reducing process steps	Selectivity results in loss of co-extracted minor polyphenols, potentially eliminating polyvalent bioactivity profiles and synergistic compound interaction ; screening individual extracts against multiple XAD MPRs necessary due to system complexity ; larger molecules (proteins, lignocellulosic material) physically excluded based on size ; polar compounds pass through without interaction; resin type and polarity matching critical (e.g., acrylic XAD7HP exhibits stronger affinity for flavonoids than non-polar XAD4) ; surface area and pore diameter variations reported (∼10 % across literature)	(Alam et al., 2021; Aljawarneh et al., 2024; Che Zain et al., 2020; Della Posta et al., 2022; DuPont, 2025; Jakovljević Kovač et al., 2021; Seif Zadeh & Zeppa, 2022; Sobhani et al., 2023); Table 4

## 4 Analysis of polyphenols

### 4.1 Absorption spectroscopy assays

Colorimetric and spectrophotometric methods enable rapid quantification of total polyphenol contents from food, food waste, and plant extracts. These approaches are practical, cost-effective, and widely adopted for rapid screening of multiple samples, as they determine the total polyphenol concentration without separating individual compounds. Total phenolic content is typically calculated as an equivalent of a standard gallic acid calibration curve and expressed in milligrams of gallic acid equivalent (GAE) per gram of extract. Total flavonoid content is quantified using aluminum chloride colorimetric assay with quercetin or rutin as standard reference compounds and expressed in milligrams of quercetin equivalent (QE) or rutin equivalent (RE) per gram of dried plant material (Shraim et al., 2021). Condensed tannins (proanthocyanidins) are assessed using catechin as the reference standard (Guneidy et al., 2022).

### 4.2 Total phenolic content

The TPC is determined via the Folin–Ciocalteau assay, which has become a standard test for estimating the polyphenol concentration in extracts. Although the specific reaction mechanism behind the assay remains unknown, the use of electrons in an alkaline medium from phenolic compounds to a phosphotungstic/phosphomolybdenum complex is suggested. The Folin–Ciocalteu reagent oxidizes phenolic compounds to produce a soluble blue complex with a maximum absorbance at 760 nm whose intensity correlates with antioxidant concentration. However, the precise structure and reaction mechanism of this blue chromophore have not been fully elucidated (Samara et al., 2022). The most common method for the assay was first described in 1965 (Singleton & Rossi, 1965), where the reaction proceeds in the dark at room temperature for 90 to 120 min. Key limitations of the Folin–Ciocalteu assay include the non-specific reactivity of the reagent toward reducing substances beyond polyphenols, including interfering compounds such as certain vitamins (ascorbic acid, vitamins A and E derivatives), reducing carbohydrates (glyceraldehyde, dihydroxyacetone), unsaturated fatty acids (arachidonic acid), and amino acids with reactive side chains (tyrosine, tryptophan, cysteine) and tyrosine-containing proteins, among others. This non-selectivity can lead to significant overestimation of total phenolic content in complex matrices, particularly in samples containing multiple interfering substances (Nowak et al., 2025; Pérez et al., 2023).

### 4.3 Total flavonoid content

The TFC can be determined from plant extracts via an assay based on the affinityof flavonoids for binding metal ions, the chelation of aluminum chloride (AlCl_3_), and the production of colored Al(III)-flavonoid complexes. This method was first introduced in 1960 and has since been widely used as a screening method in food and medicinal plant research due to its simplicity and short analysis time (Christ & Müller, 1960). Further adaptations involve the use of potassium acetate (C_2_H_3_KO_2_), sodium nitrite (NaNO_2_) or sodium hydroxide (NaOH) in the assay to adjust the color (Pękal & Pyrzynska, 2014). The aluminum chloride colorimetric assay for total flavonoid content has faced considerable criticism due to fundamental limitations: different flavonoid subclasses form Al(III) complexes with distinct maximum absorbance wavelengths (λ_max_ 400–550 nm), resulting in inaccurate quantification. Many plant flavonoids do not exhibit the same absorption maximum as the standard reference compound, leading to significant false-positive (63–124 %) or false-negative (26–42 %) results, rendering reliable quantification unreliable (Shraim et al., 2021). Methods measuring absorbance at 410–430 nm selectively quantify only flavonols and flavones. Aluminum-based assays do not distinguish flavonoid subgroups and their accuracy depends on the method and reference compound used. Although simple and reproducible for individual herbal material standardization, they are unsuitable for comparing total flavonoid content across different plant materials (Struchkov et al., 2018).

### 4.4 Total tannin content

The total condensed tannin (proanthocyanidin) content can be determined using multiple complementary colorimetric assays: the vanillin–HCl assay, the butanol–HCl assay, and the 4-(dimethylamino)cinnamaldehyde (DMACA) assay, each based on the reaction of flavan-3-ol polymers with aromatic aldehydes to produce colored complexes with distinct spectrophotometric absorbance characteristics (Esquivel-Alvarado et al., 2023; Hulkko et al., 2022). The vanillin assay was first developed in the 1960s (Goldstein & Swain, 1963). This method is employed to determine catechins and proanthocyanidins from methanolic or acetonic plant extracts. The vanillin assay is specific for a narrow range of flavanols and dihydrochalcones. It is based on the reaction between vanillin and proanthocyanidins in the presence of an acid catalyst, which results in the formation of a red-colored complex with a maximum absorbance at 500 nm at 25–30 °C (Sarkar & Howarth, 1976; Schofield et al., 2001; Sun et al., 1998). In addition to the vanillin assay, an optimized method utilizing DMACA and hydrogen chloride (HCl) has gained popularity because of its increased sensitivity (Li et al., 1996). The reaction products of proanthocyanidins and DMACA exhibit maximum absorbance at 643 nm at room temperature after 20–30 min of incubation. A key advantage of DMACA is its measurement at a longer wavelength (640 nm), which produces a bathochromic shift from the natural Proanthocyanidin (PAC) absorbance at 260 nm. This wavelength shift effectively minimizes potential interference from red-colored anthocyanins and other phytochemicals. However, DMACA has notable limitations: (i) flavan-3-ols (catechins and epicatechins) and certain anthocyanins (cyanidins and delphinidins) can also react with DMACA, causing minor interference; (ii) the chemical reaction mechanism producing the 640 nm color shift remains incompletely understood; and (iii) PAC polymerization degree affects reactivity, with PACs containing more than 10 monomer units showing variable detection efficiency (Mannino et al., 2021).

The total hydrolysable tannin content can be determined by calculating the difference in TPC measured before and after selective precipitation of tannins using complexing agents (casein or polyvinylpyrrolidone), with the Folin–Ciocalteu assay applied to measure the non-precipitated phenolic compounds remaining in the supernatant. However, this indirect method has significant limitations: casein and polyvinylpyrrolidone cause unspecific precipitation, removing not only condensed tannin polymers but also monomeric flavonoid units, resulting in inaccurate quantification of true hydrolysable tannin content and leading to false-negative results (Martins et al., 2021). Via the ability to form insoluble complexes with polyvinylpolypyrrolidone, hydrolyzable tannins can be removed from hydroalcoholic extracts by centrifugation, yielding a tannin-free supernatant (Castagna et al., 2022).

## 5 Pharmaceutical application of polyphenols

Polyphenols are classified as pan-assay interference compounds (PAINS), reflecting their propensity to form nonspecific interactions as ligands with diverse protein and enzyme targets, thereby exhibiting promiscuous binding behavior that lacks the selectivity characteristic of many conventional pharmaceutical drugs. This promiscuous inhibition operates through two primary mechanisms: (i) non-specific binding to protein/enzyme targets via multiple hydrogen-bonding donors and acceptors, and (ii) colloidal aggregation forming spherical aggregates that nonspecifically interact with proteins (Lešnik et al., 2025). However, this promiscuity has potential therapeutic relevance: during inflammatory viral infections such as COVID-19, polyphenol aglycones (generated through β-glucuronidase release from neutrophils and macrophages) exhibit broad-spectrum antiviral activity through promiscuous binding to diverse virion surfaces and intracellular viral particles, spanning multiple virus genera including SARS-CoV-2. Polyphenols such as luteolin, quercetin, baicalein, curcumin, and fisetin have demonstrated reduced virion replication in pre-clinical studies, suggesting that spatial selectivity of polyphenolic activation to sites of pathogenic infection may provide a therapeutic avenue for natural product antivirals against diverse infectious diseases (Sheridan & Spelman, 2022).

Furthermore, polyphenols have been shown to exert synergistic effects with other naturally occurring or synthetic compounds in various disease contexts, as previously discussed for curcumin–quercetin combinations enhancing antiproliferative and pro-apoptotic effects in multiple cancer cell lines (Srivastava & Srivastava, 2019), for quercetin co-administered with remdesivir or favipiravir to improve clinical outcomes and reduce inflammatory markers in severe COVID-19 patients (Shohan et al., 2022), and for keracyanin combined with oleic acid demonstrating increased anti-inflammatory potential through enhanced inhibition of pro-inflammatory cytokine production (Santamarina et al., 2021). These synergistic effects suggest a broader role for polyphenols in modern medicine, including their application in dietary recommendations, nutraceuticals, or as primary therapeutic agents in treatments.

### 5.1 Nutraceutical application of polyphenols

Interest in the use of polyphenols as nutraceuticals has increased in recent years, particularly concerning their potential benefits for managing gastrointestinal dysfunctions, such as irritable bowel syndrome (IBS), inflammatory bowel diseases (IBDs), ulcerative colitis, and Crohn's disease. The mechanisms by which polyphenols influence gut health and overall systemic health include the modulation of the gut microbiota, reduced inflammation, and alleviation of oxidative stress (Alves-Santos et al., 2020; Catalkaya et al., 2020). Proanthocyanins stimulate mucus production and support the function of the intestinal barrier, which is the primary protective barrier of the mucosal tissue of the gut (Alves-Santos et al., 2020; Catalkaya et al., 2020; Dey et al., 2019; Luo et al., 2019).

The composition and functional capacity of the gut microbiota have been linked to multiple aspects of health and disease, including inflammatory processes, metabolic homeostasis, and carcinogenesis. Dysbiosis, alterations in microbial diversity and composition, is associated with obesity, type 2 diabetes, inflammatory bowel disease, and cancer, through mechanisms involving reduced short-chain fatty acid-producing bacteria and enrichment of pro-inflammatory species, thereby promoting increased intestinal permeability and systemic inflammation (de Vos et al., 2022; Sultan et al., 2021). Currently, numerous studies have demonstrated that polyphenol intake affects the gut microbiota composition through selective prebiotic effects, enhancing the growth of beneficial bacterial strains, as well as antimicrobial effects against pathogenic bacteria (Alves-Santos et al., 2020; Catalkaya et al., 2020; Mithul Aravind et al., 2021). The consumption of berry supplements, typically characterized by high concentrations of anthocyanidins, tannins, stilbenes, and phenolic acids, has been shown to modulate colonic bacteria in human and animal trials (Lavefve et al., 2020). Moreover, it was previously reported that cloudberry polyphenol intake modulates the gut microbiota and immune response in mice, leading to a smaller adenoma diameter (Päivärinta et al., 2016). In human hosts, polyphenol-rich diets have been linked to increased *Bifidobacterium*, *Bacteroides*, *Lactobacillus*, *Roseburia*, and *Akkermansia* colonization in the gut, as well as a decreased *Firmicutes* to *Bacteroides* ratio, which is commonly used as a marker for gut microbiota health (Alves-Santos et al., 2020; Mithul Aravind et al., 2021).

With respect to IBS, *Mentha × piperita* oil, extracts from *Aloe barbadensis* and *Cynara cardunculus* var. *scolymus*, and the polyphenol group of curcumins have been studied in clinical settings with promising results, including reduced gut sensitivity, colonic spasms, and abdominal pain (Ahluwalia et al., 2020; Chiarioni et al., 2023; Roudsari et al., 2019). These supplements have not shown severe adverse effects in human intervention studies; however, mild gastrointestinal distress is common (Giang et al., 2022). Previous reviews have highlighted individual polyphenols that modulate intestinal immune responses and attenuate intestinal inflammation in vivo, including curcumin (which improves intestinal barrier function by modulating tight junction organization), quercetin and its glycosides quercitrin and rutin (which inhibit NF-κB and MAPK signaling pathways), and various catechins such as epigallocatechin-3-gallate (EGCG) and epicatechin (which maintain microbiota composition and reduce inflammatory cytokine production). These polyphenols exert immunomodulatory effects through multiple mechanisms: inhibition of lipopolysaccharide-induced NF-κB p65 translocation in dendritic cells, reduction of pro-inflammatory cytokines (TNF-α, IL-6, IL-1β), enhancement of regulatory T cell differentiation, and promotion of short-chain fatty acid-producing bacteria enrichment, collectively contributing to improved intestinal homeostasis and reduced systemic inflammation (Bao et al., 2019; Jamieson et al., 2023; Shabbir et al., 2021). In addition, it was reported that resveratrol intake ameliorated colitis symptoms and colon tissue damage in mouse models by decreasing colon inflammation through the regulation of proinflammatory cytokines (Li, Han, et al., 2020). Another investigation found that green tea polyphenols, which are rich in catechins, decreased the expression of pro-inflammatory cytokines and mitigated the adverse effects of a high-fat diet on the gut microbiota in a canine model (Li, Rahman, et al., 2020). Overall, curcumin as well as green tea polyphenols have shown promising results in human intervention studies for mild-to-moderate ulcerative colitis (Hagan et al., 2021; Sadeghi et al., 2020). Resveratrol, a plant-derived polyphenolic compound, demonstrated significant therapeutic efficacy in preclinical IBD models especially at doses exceeding 80 mg/kg, reducing histopathological injury and intestinal mucosal damage. This efficacy is mediated through immune modulation and antioxidant activity (Gu et al., 2024).

Nevertheless, only a few observational studies have been conducted to evaluate the effects of habitual dietary polyphenol intake on patients with IBS and IBD; however, a lack of detailed information on polyphenol intake has made it difficult to draw definite conclusions (Hagan et al., 2021). Moreover, given the wide range of polyphenolic supplements currently marketed, concerns have been raised regarding the potential adverse effects of supplement formulations and their additives on disease management and consumer health. Polyphenol supplements in purified form can exert harmful effects through multiple mechanisms: (i) inhibition of digestive enzymes (amylase, protease, lipase), reducing nutrient bioavailability and causing gastrointestinal dysfunction; (ii) iron sequestration leading to iron deficiency anemia in susceptible populations; (iii) adverse drug interactions through cytochrome P450 (CYP3A4) inhibition, altering drug metabolism and bioavailability; and (iv) microbiota dysbiosis through selective inhibition of beneficial bacteria (*Lactobacillus*, *Bifidobacterium*) and enrichment of pathogenic species. Furthermore, quality control issues in the supplement industry, including adulteration with undeclared active pharmaceutical ingredients, heavy metal contamination, pesticide residues, and mislabeled product composition, pose significant health risks that may exceed the therapeutic benefit of the declared polyphenolic compounds (Djaoudene et al., 2023; Duda-Chodak & Tarko, 2023; Golonka et al., 2019).

### 5.2 Drug-likeness of polyphenols

The drug-likeness properties of the polyphenols in this study were evaluated on the basis of Lipinski's rule of five. This rule outlines the following parameters: i) molecular weight (MW) ≤ 500 Da, ii) number of hydrogen bond donors (HDB) ≤ 5, iii) number of hydrogen bond acceptors (HBA) ≤ 10, and iv) octanol/water partition coefficient (logP) < 5. Notably, all the parameters are multiples of five; hence, the name (Lipinski et al., 2012). Beyond these core parameters, an additional criterion commonly evaluated is v) rotatable bonds <10 (Veber et al., 2002). These rules are purely phenomenological and do not definitively guarantee or reject a polyphenol's suitability for in vivo drug research. For example, despite multiple Lipinski rule violations, such as epigallocatechin-3-gallate (EGCG with two violations) and theaflavin-3,3′-digallate (TF3 with three violations), resulting in low systemic bioavailability, these polyphenols demonstrate significant biological activity, suggesting that mechanisms beyond systemic absorption contribute to their therapeutic effects, including local intestinal activity, microbiota-mediated metabolism, or active metabolite generation (Mhatre et al., 2021; Zhou et al., 2025).

It has been reported that these molecules undergo extensive anaerobic fermentation by human fecal microbiota, generating bioactive metabolites including hydroxylated phenylcarboxylic acids, phenylpropionic acids, and theanaphthoquinone. These microbial metabolites, combined with the direct immunomodulatory effects of the parent compounds on colonic bacteria, promote the growth of beneficial commensal bacteria (*Bacteroides*, *Faecalibacterium*, *Bifidobacterium*, *Parabacteroides*) while inhibiting pathogenic species (*Prevotella*, *Fusobacterium*), thereby mediating their therapeutic effects through local intestinal activity and microbiota-derived signals rather than systemic absorption (da Pinaffi-Langley et al., 2024; Lippolis et al., 2023; Liu et al., 2021).

Table A.11 in the supplementary material presents 169 selected polyphenols reviewed in this study and their Lipinski parameters, indicating their suitability as drug candidates. Generally, most polyphenols exhibit LogP values lower than 5 due to the high number of hydroxyl groups on their nonpolar backbones, with only 3.0 % being hydrophobic enough to have a LogP greater than 5. Table A.11 shows that 52.1 % of the evaluated polyphenols had 0 violations, 10.1 % had 1 violation, 18.3 % had 2 violations, 18.3 % had 3 violations, and 0.4 % had 4 or 5 violations. This generally indicates good drug-likeness of polyphenols as a group; however, tannins were observed to cause more Lipinski violations. Polyphenols, especially those with high molecular weights, tend to have poor bioavailability, which may be a limiting factor for their efficacy. Only 5–10 % of polyphenols are absorbed in the small intestine, while the remaining 90–95 % accumulate in the large intestine and are extensively metabolized by the resident gut microbiota into bioactive metabolites including phenolic acids, phenylpropionates, and other small molecular phenolic compounds that can be subsequently absorbed or exert local effects on intestinal health (Long et al., 2021).

Diets high in dietary fiber have also been shown to influence polyphenol accessibility (Catalkaya et al., 2020; Jakobek & Matić, 2019). It has been suggested that some of the health benefits of a polyphenol-rich mixture may be associated with the use of absorbable low-molecular-weight bacterial polyphenol metabolites rather than the original polyphenol itself (Catalkaya et al., 2020; Mithul Aravind et al., 2021). Polyphenols are biotransformed when they are metabolized by microorganisms in the intestines of humans and animals (Lavefve et al., 2020; Sharma et al., 2022). A previous investigation reported the biotransformation of complex polyphenols from various edible nuts into smaller polyphenols via microbial digestion (Rocchetti et al., 2019). Owing to these qualitative and quantitative changes in compound profiles, fermentation of polyphenol-rich food before consumption has also been investigated to improve polyphenol bioavailability and enhance health benefits (Sharma et al., 2022).

When foods containing polyphenols are ingested and absorped by humans and animals, the high microbial activity in the gastrointestinal tract has been shown to catalyze the cleavage of many large polyphenols into smaller compounds that, in turn, exhibit fewer violations of Lipinski's rule of five. Previously a review investigated the bioavailability of polyphenols and reported that ingested foods containing free polyphenols with several Lipinski violations are presumed to degrade through dehydration reactions to hydroxybenzoic acids via gastric absorption, as the gastrointestinal tract possesses high bacterial conjugative enzyme activity (Chen et al., 2019).

### 5.3 Polyphenols as ligands

Polyphenols, proteins, and enzymes are found in all plants and can form protein–polyphenol or enzyme–polyphenol complexes. In this review, enzyme–polyphenol complexes are referred to as protein–polyphenol complexes. Not all polyphenols in biomass form these complexes with proteins and enzymes; however, as free compounds in an extract, polyphenols are known to interact with proteins and enzymes after liberation from the biomass structure. Extensive matrices of protein–polyphenol complexes can form through reversible non-covalent interactions (hydrogen bonds, van der Waals forces, hydrophobic and electrostatic interactions) or irreversible covalent bonds (Michael addition of quinone metabolites to nucleophilic amino acids). These complex matrices trap and enclose polyphenols within the protein structure, restricting access to the polyphenol binding sites responsible for redox cycling and antioxidant activity. Consequently, when polyphenols are complexed with proteins, their antioxidant capacity is significantly reduced through ‘masking’ of active hydroxyl groups. However, this enclosure can paradoxically provide protection during gastrointestinal transit, allowing controlled release of polyphenols during digestion and potential regeneration of antioxidant activity in the intestinal tract (Feng et al., 2023; Quan et al., 2019).

An example of a strong bond between a protein and a polyphenol is the interaction of quercetin with the SARS-CoV-2 spike protein TMPRSS2. Using molecular docking, a recent investigation found a high molecular bonding energy between the two, which suggests that quercetin has the potential to inactivate the protein and block viral cell entry in vivo (Manjunathan et al., 2022).

Previous findings underscore the complexity of polyphenol-protein interactions, which encompass diverse noncovalent mechanisms including hydrophobic effects, hydrogen bonding—both direct and water-mediated—alongside π-stacking interactions, though their relative importance varies across polyphenolic classes. Notably, molecular dynamics simulations highlight water-mediated hydrogen bonds as fundamental to polyphenol binding, revealing dynamic binding patterns that static crystal structures alone cannot adequately capture. Glycosylation emerges as a regulatory factor, modulating interactions through the formation of intricate water networks at the binding interface. Crucially, polyphenols differ fundamentally from conventional synthetic drugs in their binding behavior: rather than exhibiting the stable, specific binding modes typical of pharmaceutical ligands, polyphenols display inherent flexibility and promiscuity driven by their propensity for water-mediated interactions. Since these enzymatic reactions are highly complex and multiple interactions can occur simultaneously, combining molecular docking results with in vitro assays of polyphenols is crucial for understanding individual inhibition mechanisms and harness their therapeutic applications (Fredsgaard, Kaniki, et al., 2023; Lešnik et al., 2025).

Direct hydrogen bonding between polyphenolic hydroxyl groups and amino acid residues such as serine, tyrosine, and glutamate constitutes a primary stabilizing force, particularly in flavonoid-protein complexes where quercetin and catechin bind to transcription factor FOXO3 through hydrogen bonding and hydrophobic interactions at distinct sites, thereby modulating IKKα transcription and exhibiting enhanced antioxidant effects against oxidative stress-induced liver injury (Guan et al., 2025). Hydrophobic interactions involving aromatic rings complement hydrogen bonding networks and are prevalent across all polyphenol classes, with π-stacking interactions with phenylalanine, tryptophan, and histidine residues providing additional stabilization through face-to-face or T-shaped geometries (Lešnik et al., 2025). Covalent interactions form for example during food processing through oxidation of polyphenols to quinone intermediates, which subsequently react with nucleophilic amino acid residues including lysine, cysteine, and tryptophan, resulting in enhanced antioxidant capacity and altered protein structure that can reduce allergenicity, as demonstrated by enzymatic cross-linking of epigallocatechin gallate (EGCG) to lactoferrin, thereby reducing protein allergenicity while maintaining bioactivity (Zhang et al., 2024).

Metal coordination through catechol and gallol hydroxyl groups represents a specialized binding mechanism, particularly in phenolic acids and flavonoids that chelate iron, copper, and zinc ions, enabling therapeutic effects in metalloprotein-dependent pathways (Lešnik et al., 2025). Collectively, these non-covalent and covalent mechanisms demonstrate that polyphenols function as versatile molecular modulators capable of suppressing pathogenic pathways and enhancing cellular defense mechanisms through multivalent protein interactions.

Selected polyphenols have been reviewed for their potential medicinal applications and cytotoxicities (the ability of a substance to cause damage to cells), as shown in Table 7. Bioactivities refer to the list of bioactivities #1–21.

**Table 7:** – Selected polyphenols reviewed for their in vitro and in vivo nonantiviral potential bioactive properties in humans and their cytotoxicities. The polyphenols are sorted by molecular weight. *: Cell proliferation assay. **: 3-(4,5-Dimethylthiazol-2-yl)-2,5-diphenyl diphenyltetrazolium bromide (MTT) assay using mock-infected Madin-Darby canine kidney (MDCK) cells. Bioactivity: Refers to the list of bioactivities #1–21 below the table. ND: No data.

**Polyphenol**	**Bioactivity**	**Cytotoxicity CC**_**50**_	**Ref.**
Caffeic acid	1, 4, 8, 10, 12, 14	> 555 μM**	(Bailly & Cotelle, 2009; Jalali et al., 2021; Magnani et al., 2014; Mehrotra et al., 2011; Rajendra Prasad et al., 2011; Serafim et al., 2011)
Ferulic acid	1, 4, 5, 8, 14, 18	702 μM*	(Hariono et al., 2016; Jalali et al., 2021; Ma et al., 2020; Ou & Kwok, 2004; Serafim et al., 2011)
Chrysin	1, 2, 4, 5, 9, 11, 15, 17, 20	393 μM**,	(Basu et al., 2016; Falbo & Aiello, 2023; Kasala et al., 2015; Ohkura et al., 2020)
Pinocembrin	1, 5, 8, 9, 20	ND	(Chen et al., 2013; Estevinho et al., 2008; Feng et al., 2012; Kumar et al., 2007; Pan et al., 2011; Rasul et al., 2013; Žižić et al., 2013)
Apigenin	1, 5, 7, 9, 12, 14, 15, 20	247 ± 23 μM**	(Al Shaal et al., 2011; Jalali et al., 2021; Salehi et al., 2019)
Kaempferol	1, 5, 7, 8, 11, 12, 14, 15, 17, 20	> 349 μM**	(Bangar et al., 2023; Holland et al., 2023; Jalali et al., 2021)
Quercetin	1, 2, 3, 5, 9, 14, 15, 17, 20	480 μM*, 313 ± 23 μM**	(Agrawal et al., 2020; Holland et al., 2023; Jalali et al., 2021; Kaihatsu et al., 2014; Larson et al., 2012; Mulvihill & Huff, 2010; Murakami et al., 2008; Salaritabar et al., 2017; Wang et al., 2016)
Isorhamnetin	1, 2, 4, 5, 7, 9, 14, 17, 20	>280 μM**	(Abdal Dayem et al., 2015; Gong et al., 2020; Jaramillo et al., 2010; Mulvihill & Huff, 2010; Wang et al., 2016)
Myricetin	1, 8, 9, 10, 14, 15, 16, 17, 20	505 μM*, 749 ± 12 μM**	(Kaihatsu et al., 2014; Ma et al., 2015; Park et al., 2016; Sang et al., 2021; Taheri et al., 2020)
Chlorogenic acid	1, 5, 8, 9, 12, 14, 15, 17, 21	364 μM**	(Bailly & Cotelle, 2009; Ding et al., 2017; Jalali et al., 2021; Naveed et al., 2018; Wang et al., 2009; Zhou et al., 2017)
Isoquercitrin	4, 6, 9, 11, 15, 16	977 μM*	(Alvarez et al., 2012; Kim et al., 2010; Valentová et al., 2014; Wang et al., 2016; Zwicker et al., 2019)
Myricitrin	1, 2, 5, 10, 19, 20	> 224 μM**	(Córdova et al., 2011; Huang et al., 2014; Kassem et al., 2016; Motlhatlego et al., 2021; Xu et al., 2013; Yan et al., 2017)
Cynaroside	1, 5, 11, 14, 15, 17	ND	(Bouyahya et al., 2023; Hazra et al., 2022; Kim et al., 2013; Park et al., 2002; Žemlička et al., 2014)
Kuromanin	1, 11, 15, 17, 20	ND	(Cásedas et al., 2019; Kamei et al., 1995; Moghadam et al., 2023; Mughairbi et al., 2023)
Keracyanin	1, 5, 11, 15, 17, 20	ND	(Adisakwattana et al., 2011; Bishayee et al., 2010; Chen et al., 2006; Santamarina et al., 2021; Shimazu et al., 2021)

The pharmaceutical effects of the selected polyphenols shown in Table 7 are follows:

(1) Anticarcinogenic: Preventing the development of cancer.
(2) Antiatherogenic: Preventing artery lesions.
(3) Antiulcer: Preventing sores on the lining of the stomach and small intestines. This often requires a colon-specific drug delivery system, such as an encapsulated form.
(4) Antithrombotic and Anticoagulant: Preventing the formation of blood clots.
(5) Anti-inflammatory: Preventing inflammation or swelling.
(6) Antiallergenic: Preventing allergic reactions.
(7) Immune-modulating: Changing immune system mechanisms.
(8) Antimicrobial: Preventing or killing pathogenic microorganisms.
(9) Vasodilatory/Antihypertension: Widening the blood vessels, thereby decreasing blood pressure.
(10) Analgesic activities: Possessing pain-relieving properties.
(11) Antihepatotoxicity: Preventing liver damage.
(12) Antityrosinase/Skin Damage Limiting: Promoting skin whitening, limiting melanin production, or providing skin protection.
(13) Anti-osteoporosis: Reducing or treating osteoporosis to decrease the risk of fracture.
(14) Antidepressant: Mood-regulating properties.
(15) Antidiabetic: Regulating glucose levels and insulin activity.
(16) Chemoprotective: Protecting healthy tissue from side effects caused by certain anticancer drugs.
(17) Antiobesity: Exhibiting weight-loss effects or contributing to weight control.
(18) Anti-infertility (male): Increasing sperm viability.
(19) Fertility regulation (female): Inducing anovulation for birth control.
(20) Antidementia/Neuroprotective: Exhibiting protective effects that reduce memory dysfunction.
(21) Antipyretic: Fever-reducing.

Many of these effects could be interchangeable and, therefore, linked. Clear examples include #4 (Antithrombotic and Anticoagulant) with #9 (Vasodilatory/Antihypertension) and #15 (Antidiabetic) with #17 (Antiobesity), while others are indirectly correlated.

Polyphenols have multiple inhibitory effects on enzymes such as aldose reductase, xanthine oxidase, cyclooxygenase-2, phosphodiesterase, adenosine deaminase, and lipoxygenase (Chu, 2022; Maradesha et al., 2022; Scanu et al., 2022). This broad inhibition spectrum may be responsible for preventing or mitigating conditions like diabetes, hyperuricemia leading to gout, rheumatoid arthritis, the production of prostaglandins associated with pain and inflammation, hypertension and other cardiovascular diseases, and the development and progression of neurodegenerative diseases and cancer (Arias-Sánchez et al., 2023; Aryal et al., 2024; Hedayati et al., 2023; Iqbal et al., 2023; Patra et al., 2021; Rubín-García et al., 2022; Scanu et al., 2022; Wang et al., 2022).

Table 8 presents the literature regarding the use of polyphenols as ligands for enzymes involved in pathogenic pathways.

**Table 8:** – Enzymes inhibited or regulated by polyphenols and their inhibition activities. IC_50_: Half maximal inhibitory concentration; EC_50_: Half maximal effective concentration. SERCA: Sarcoplasmic/endoplasmic reticulum Ca^2+^-ATPase. COX-2: Cyclooxygenase-2.

**Enzyme inhibited**	**Ligand**	**Inhibition activity**	**Ref.**
Adenosine deaminase	Hyperoside	Docking energy: − 46.6 ± 8.3 kcal/mol	(Uba et al., 2023)
	Chlorogenic acid	Docking energy: − 18.8 ± 4.6 kcal/mol	
	Curcumin	Significantly decreased levels of Adenosine deaminase at 12.5 mg/kg curcumin in rats with Cadmium-induced episodic memory loss	(Akinyemi et al., 2017)
	Epigallocatechin gallate	IC_50_ inhibition:106.7 μM	(Arun et al., 2019)
		Docking energy: − 6.9 kcal/mol	
	Genistein	IC_50_ inhibition: 1480.2 μM	(Ni et al., 2016)
	Keracyanin	IC_50_ inhibition: 1007.5 μM	
	Quercetin	Docking energy: − 8.9 ± 7.3 kcal/mol	(Agrawal, 2011; Uba et al., 2023)
Aldose reductase	Isorhamnetin-3-glucoside	IC_50_ inhibition: 1.4 μM	(Lee et al., 2005)
	Chlorogenic acid	IC_50_ inhibition: 5.5 μM	(Alim et al., 2017)
		Docking energy: − 8.0 kcal/mol	(Purnamasari et al., 2020)
	Neochlorogenic acid	Docking energy: − 8.3 kcal/mol	
	Acacetin	IC_50_ inhibition: 4.8 μM	(Manivannan et al., 2015)
		Docking energy: − 10.0 kcal/mol	
	Ferulic acid	Docking energy: − 7.2 kcal/mol	
		Calculated IC_50_ inhibition: 5.18 μM	
		IC_50_ inhibition: 21.9 μM	
		IC_50_ inhibition: 69 μM	(Alim et al., 2017)
	Sinapic acid	IC_50_ inhibition: 33 μM	
	Tannic acid	IC_50_ inhibition: 0.5 μM	
	Epigallocatechin gallate	Docking energy: − 17.0 kcal/mol	(Balestri et al., 2020)
Ca^2+^-ATPase	Quercetin	IC_50_ inhibition: 80.0 μM	(Roufogalis et al., 1999; Wüthrich & Schatzmann, 1980)
	Gossypol	IC_50_ inhibition: 13.0 μM	(Kanwar et al., 1989; Roufogalis et al., 1999)
	Myricetin	IC_50_ inhibition: 6.0 μM	(Roufogalis et al., 1999; Thiyagarajah et al., 1991)
		Significantly lower apoptosis in rats fed with high glucose diets by regulation of SERCA at 20 μM	(Karunakaran et al., 2019)
	Epigallocatechin gallate	Docking energy: − 8.3 kcal/mol	(Rinaldi et al., 2021)
	6-Gingerol	EC_50_ of 1.8 ± 0.3 μM on SERCA	(Sfragano et al., 2023)
	Curcumin	IC_50_ inhibition of ovarian cancer in MDAH2774 (p53-null type) cell line: 7.0 μM	(Seo et al., 2016)
		IC_50_ inhibition of ovarian cancer in SKOV3 (p53-mutant type) cell line: 1.3 μM	
		IC_50_ inhibition of ovarian cancer in PA1 (p53-wild type) cell line: 6.5 μM	
	Baicalein	Significant increased cardioprotective effects in rats with heart failure at 100 mg/kg	(Zhao et al., 2016)
Cyclooxygenase-2	Epigallocatechin-3-gallate	Decreased cell growth and apoptosis in vitro on LNCaP human prostate cancer cells at 10 μM	(Bouyahya et al., 2023)
		Decreased cell growth and apoptosis in vitro on PC-3 human prostate cancer cells at 10 μM	
		Decreased cell growth and apoptosis in vitro on CWR22R*v*1 human prostate cancer cells at 10 μM	
		Decreased mortality in CWR22R*v*1 human prostate cancer-induced athymic nude mice with 1 ppt epigallocatechin-3-gallate water	
	Syringaldehyde	IC_50_ inhibition: 192.1 μM	(Stanikunaite et al., 2009)
	Syringic acid	IC_50_ inhibition: 20.2 μM	
	Ellagic acid	Inhibition of COX-2 in rats at 60 mg/kg with 1,2-dimethylhydrazine-induced colon carcinogenesis	(Umesalma & Sudhandiran, 2010)
	Quercetin	Significantly decreased COX-2 expression in vitro in recombinant human IL-1β cytokine at 100 μM	(Sung et al., 2012)
	Myricetin	Blocked lipoteichoic acid-induced COX-2 expression in vitro in human gingival fibroblast at 10 μM	(Gutiérrez-Venegas et al., 2014)
		Docking energy: − 8.9 kcal/mol	(Dash et al., 2015)
	5-Deoxykaempferol	Docking energy: − 9.1 kcal/mol	
	Equol	Docking energy: − 9.9 kcal/mol	
	Demethyltexasin	Docking energy: − 9.9 kcal/mol	
	Eriodictyol	Docking energy: − 9.5 kcal/mol	
	Quercetin	Docking energy: − 8.9 kcal/mol	
	3’-Hydroxydaidzein	Docking energy: − 9.9 kcal/mol	
	Quercetin-3-methyl ether	Docking energy: − 8.3 kcal/mol	
	Kaempferol	Docking energy: − 10.6 kcal/mol	
	Delphinidin	Docking energy: − 8.9 kcal/mol	
	Luteolin	Docking energy: − 10.7 kcal/mol	
	Isorhamnetin	Docking energy: − 8.2 kcal/mol	
Phosphodiesterase	Tricin	Docking energy: − 6.8 kcal/mol	(Madeswaran et al., 2012)
		Calculated IC_50_ inhibition: 10.1 μM	
	Tricetin	Docking energy: − 7.5 kcal/mol	
		Calculated IC_50_ inhibition: 3.2 μM	
	Diosmetin	Docking energy: − 6.8 kcal/mol	
		Calculated IC_50_ inhibition: 10.7 μM	
		IC_50_ inhibition: 14.4 ± 6.2 μM	(Ko et al., 2004)
	Luteolin	IC_50_ inhibition: 21.5 ± 2.9 μM	
	Cynaroside	IC_50_ inhibition: >100 μM	
	Genistein	IC_50_ inhibition: 16.8 ± 2.3 μM	
	Quercetin	IC_50_ inhibition: 27.8 ± 5.7 μM	
	Myricetin	IC_50_ inhibition: 24.9 ± 3.6 μM	
	1,3,5-trihydroxy-4-(3-hydroxy-methylbutyl)xanthone	IC_50_ inhibition: 3.2 ± 0.2 μM	(Sabphon et al., 2015)
	1,3,5-trihydroxy-4-prenylxanthone	IC_50_ inhibition: 3.0 ± 1.7 μM	
Lipoxygenase	Catechin	Docking energy: − 8.1 kcal/mol	(Rajak et al., 2023)
	Epicatechin gallate	Docking energy: − 9.1 kcal/mol	
	Epigallocatechin	Docking energy: − 7.8 kcal/mol	
	Epigallocatechin gallate	Docking energy: − 9.6 kcal/mol	
	Gallocatechin	Docking energy: − 8.3 kcal/mol	
	Epicatechin	Docking energy: − 9.5 kcal/mol	
		IC_50_ inhibition: 60 μM	(Sadik et al., 2003)
	Genistein	IC_50_ inhibition: 18 μM	
	Baicalein	IC_50_ inhibition: 1 μM	
	Luteolin	IC_50_ inhibition: 0.6 μM	
	Fisetin	IC_50_ inhibition: 1.8 μM	
	Myricetin	IC_50_ inhibition: 18 μM	
	Morin	IC_50_ inhibition: 6 μM	
	Kaempferol	IC_50_ inhibition: 15 μM	
	Galagin	IC_50_ inhibition: 45 μM	
	Taxifolin	IC_50_ inhibition: 25 μM	
	Hesperetin	IC_50_ inhibition: 90 μM	
	Quercetin	IC_50_ inhibition: 4 μM	
		IC_50_ inhibition: 7.6 ± 0.3 μM	(Bertanha et al., 2020)
	Rosmarinic acid	Docking energy: − 7.6 kcal/mol	(Chai et al., 2023)
Xanthine oxidase	Quercetin	Docking energy: − 6.8 kcal/mol	(Hendriani et al., 2017)
		Calculated IC_50_ inhibition: 2.2 μM	
		IC_50_ inhibition: 14.5 μM	
		IC_50_ inhibition: 0.44 μM	(Nagao et al., 1999)
	Kaempferol	IC_50_ inhibition: 0.67 μM	
	Chrysin	IC_50_ inhibition: 5.0 μM	
	Luteolin	IC_50_ inhibition: 0.96 μM	
	Myricetin	IC_50_ inhibition: 1.3 μM	
	Rhamnetin	IC_50_ inhibition: >50 μM	
	Isorhamnetin	IC_50_ inhibition: 0.40 μM	
	Tangeretin	IC_50_ inhibition: >100 μM	
	Rutin	IC_50_ inhibition: 46.8 μM	
	Genistein	IC_50_ inhibition: 83.0 μM	
	Phloretin	IC_50_ inhibition: 0.66 μM	
	Apigenidin	IC_50_ inhibition: 29.1 μM	
	Pelargonidin	IC_50_ inhibition: 21.9 μM	
	Cyanidin	IC_50_ inhibition: 27.8 μM	

As seen from Table 8, the most promising polyphenols that exhibit inhibitory effects against enzymes involved in pathogenic pathways include hyperoside, curcumin, and quercetin for adenosine deaminase; isorhamnetin-3-glucoside, tannic acid, epigallocatechin, and acacetin for aldose reductase; myricetin and 6-gingerol for Ca^2+^-ATPase; luteolin and keampferol for cyclooxygenase-2; tricetin, 1,3,5-trihydroxy-4-(3-hydroxy-methylbutyl)xanthone, and 1,3,5-trihydroxy-4-prenylxanthone for phosphodiesterase; luteolin, morin, and quercetin for lipoxygenase; and quercetin, kaempferol, luteolin, isorhamnetin, and phloretin for xanthine oxidase. Table 9 Table 9 reviews the multiple diseases and disorders associated with these enzymes.

**Table 9:** – Enzymes inhibited or regulated by polyphenols and their nonviral induced or associated diseases and disorders.

**Enzyme inhibited**	**Associated diseases and disorders**	**Ref.**
Adenosine deaminase	Atherosclerosis and multiple sclerosis	(Bagheri et al., 2019; Kutryb-Zajac et al., 2016; Polachini et al., 2014)
	Cancer (Bladder, breast, colorectal, gastric, leukemia, throat)	(Durak et al., 1994; Ebrahimi-Rad et al., 2018; Eroglu et al., 2000; Güleç et al., 2003; Sharma et al., 2013; Walia et al., 1995)
	Diabetes and obesity	(Chielle et al., 2015; Fulzele et al., 2015)
	Gastrointestinal diseases	(Beyazit et al., 2012; Cakal et al., 2010)
	Immunoglobulin dysregulation	(Poursharifi et al., 2009)
	Mental and neurological disorders	(Akinyemi et al., 2017; Chiba et al., 1995; Herken et al., 2006; Sasidharan et al., 2017)
Aldose reductase	Airway diseases	(Yadav et al., 2009; Yadav et al., 2011; Yadav et al., 2013)
	Cancer (Breast, colorectal, lung, prostate)	(Reddy et al., 2017; Singh et al., 2021; Srivastava et al., 2011)
	Cardiovascular diseases	(Ramana et al., 2002; Singh et al., 2021)
	Diabetes	(Dunlop, 2000; Hwang et al., 2004)
	Kidney diseases, hyperuricemia, and gout	(Dunlop, 2000; Oates, 2010)
	Sepsis	(Ramana, 2011; Srivastava et al., 2011)
Ca^2+^-ATPase	Cancer (Breast, colon, ovarian)	(Aung et al., 2007; Peters et al., 2016; Seo et al., 2016)
	Cardiovascular diseases	(Inesi et al., 2008; Periasamy et al., 2008)
	Diabetes	(Bidasee et al., 2004; Herchuelz & Pachera, 2018; Spieker et al., 1994)
	Mental and neurological disorders	(Berrocal et al., 2009; Inesi et al., 2008; Liguri et al., 1990; Mata et al., 2011)
	Muscle disorders	(Haramizu et al., 2011; Viskupicova & Rezbarikova, 2022)
Cyclooxygenase-2	Cancer (Breast, colorectal, lung, prostate)	(Dempke et al., 2001; DuBois et al., 1998; Harris et al., 2007; Wang & DuBois, 2010)
	Gastrointestinal diseases	(Mahadevan et al., 2002; Singer et al., 1998)
	Mental and neurological disorders	(Kim et al., 2015; Pasinetti & Aisen, 1998; Teismann et al., 2003)
Phosphodiesterase	Airway diseases	(Chong et al., 2017; Fan Chung, 2006; Lipworth, 2005)
	Cancer (Breast, bladder, colorectal, leukemia, lung, oral, prostate, skin)	(Engström et al., 2012; Loeb et al., 2016; Mehta & Patel, 2019; Peng et al., 2018; Quimque et al., 2021)
	Cardiovascular diseases	(Kukreja et al., 2011; Reffelmann & Kloner, 2003)
	Mental and neurological disorders	(Nabavi et al., 2019; Quimque et al., 2021)
Lipoxygenase	Atherosclerosis	(Wisastra & Dekker, 2014)
	Airway diseases	(Liu et al., 2009; Wisastra & Dekker, 2014)
	Cardiovascular diseases	(Li et al., 2013; Wisastra & Dekker, 2014)
	Gastrointestinal diseases	(Jupp et al., 2007; Stanke-Labesque et al., 2008; Wisastra & Dekker, 2014)
	Cancer (Breast, prostate, pancreatic, colorectal)	(Bhatia et al., 2003; Hennig et al., 2005; Kerjaschki et al., 2011; Suraneni et al., 2010; Wang & DuBois, 2010; Wisastra & Dekker, 2014)
Xanthine oxidase	Cancer (Bladder)	(Güleç et al., 2003)
	Cardiovascular diseases	(Berry & Hare, 2004; Dawson & Walters, 2006; Higgins et al., 2012; Polito et al., 2021)
	Kidney disease, hyperuricemia, and gout	(Dawson & Walters, 2006; Furuhashi, 2020; Polito et al., 2022)
	Liver diseases	(Battelli et al., 2001; Stirpe et al., 2002)
	Mental and neurological disorders	(Herken et al., 2006; Michel et al., 2010)

Previous research reported a significant difference in the inhibitory effects of luteolin and cynaroside (luteolin-7-glucoside) on phosphodiesterase, attributed it to the additional glucoside group, which blocked the entrance to the enzyme's active site (Ko et al., 2004). Similarly, an effect of the even more structurally similar, almost identical, isomers rhamnetin and isorhamnetin, with isorhamnetin demonstrating a > 124-fold greater inhibition of xanthine oxidase was reported (Nagao et al., 1999). Consequently, polyphenols can exhibit very similar inhibition activities despite their different internal structures, for example, anthocyanidins against xanthine oxidase.

Significant deviations were observed in the measured inhibition constants of ferulic acid against aldose reductase (Alim et al., 2017; Manivannan et al., 2015), and quercetin against lipoxygenase (Bertanha et al., 2020; Sadik et al., 2003) and xanthine oxidase (Hendriani et al., 2017; Nagao et al., 1999), as shown in Table 8.

These specific inconsistencies can be explained by factors such as different solvent systems in which inhibition assays were conducted, variations in reaction time, modifications to the assays, differences in the purities of the polyphenols and enzymes used, and whether the polyphenols were acquired as pure standards or were isolated by the researchers from biomass. The latter scenario (isolation from biomass) could lead to the coextraction of impurities that might either enhance the apparent inhibition (resulting in seemingly lower inhibition constants) or dilute the target polyphenol, causing artificially higher inhibition constants owing to a lower effective concentration of the active compound.

### 5.4 Potential adverse effects of polyphenols

Polyphenols, despite their numerous health benefits, can pose significant adverse effects and safety concerns depending on dose, chemical form, and individual health status (Duda-Chodak & Tarko, 2023). One of the most prominent issues is the inhibition of iron absorption through chelation mechanisms. Polyphenols such as catechins found in tea possess specific structural features, particularly catechol and galloyl groups, that enable them to chelate ferric and ferrous ions, substantially reducing iron bioavailability (Scarano et al., 2023). This represents a particular concern for populations at elevated risk of iron deficiency, especially those relying on plant-based diets, as polyphenol-rich foods like tea, wine, and coffee can significantly inhibit iron absorption (Živanović et al., 2025). The low bioavailability of polyphenols from dietary sources, often below 10 %, further limits their therapeutic efficacy from food alone, necessitating high-dose supplementation to achieve therapeutic concentrations (Saad et al., 2025).

Additionally, polyphenols inhibit key digestive enzymes including α-amylase and α-glucosidase through competitive binding mechanisms, potentially leading to nutrient malabsorption and altered carbohydrate digestion. Epigallocatechin gallate (EGCG) and other highly hydroxylated flavonoids show particularly strong inhibitory activity against these enzymes (Ćorković et al., 2022). Furthermore, while polyphenols can selectively enhance beneficial gut bacteria such as *Faecalibacterium* and *Akkermansia*, high-dose consumption may also suppress other bacterial populations, altering microbial balance and resulting in dysbiosis-related inflammation and metabolic dysfunction (Cao et al., 2023; Meiners et al., 2025). Polyphenol-induced changes in gut microbiota composition can consequently affect the production of short-chain fatty acids and other microbial metabolites that modulate intestinal barrier integrity and systemic inflammation (Meiners et al., 2025). Moreover, the antimicrobial properties of polyphenols demonstrate dose-dependent effects on gut microbiota composition. While polyphenols at moderate doses selectively suppress pathogenic bacteria and promote beneficial genera such as *Lactobacillus* and *Bifidobacterium*, excessive intake may reduce overall microbial diversity and inhibit commensal bacterial populations (Cano et al., 2024). This can result in altered metabolic function and intestinal barrier dysfunction (Mandal & Domb, 2024).

Polyphenol-drug interactions represent another critical safety consideration. These compounds inhibit or induce cytochrome P450 enzymes, particularly CYP3A4 and CYP2C9, as well as transporters such as P-glycoprotein, potentially causing serious interactions with anticoagulants, statins, and other medications. Additionally, isoflavonoids exhibit estrogenic activity and can inhibit thyroid peroxidase, raising concerns for hypothyroidism development, particularly in iodine-deficient or hypothyroid individuals (Duda-Chodak & Tarko, 2023).

Importantly, these adverse effects predominantly occur with high-dose supplements rather than with polyphenols consumed naturally from fruits and vegetables (Duda-Chodak & Tarko, 2023). Vulnerable populations should consult healthcare providers before supplementation. Recent bioavailability studies have demonstrated marked inter-individual variability in polyphenol absorption, with factors such as gut microbiota composition, intestinal enzyme activity, genetic polymorphisms, and dietary matrix composition significantly influencing bioavailability outcomes (Di Lorenzo et al., 2021; Saad et al., 2025). The extensive metabolism of most polyphenols into conjugated metabolites (glucuronides, sulfates, and methylated forms) and the formation of specialized molecules by gut microbiota further complicate the determination of their true biological effects in vivo (Saad et al., 2025).

## 6 Conclusion

This review examined extraction and isolation methodologies for polyphenols from food and food waste biomass, evaluated their efficacy in valorizing these sources, assessed polyphenol drug-likeness according to modified Lipinski criteria, and investigated their potential as multi-target inhibitors of pathogenic proteins and enzymes relevant to diverse disease pathways. Three advanced extraction technologies, DES, SWE, and MPR adsorption, emerged as the most promising approaches for polyphenol recovery from both conventional food and food waste matrices. DES demonstrates superior selectivity and generates multi-functional extracts with dual antioxidant and antimicrobial properties, particularly advantageous for food waste valorization, though requiring careful viscosity management and solvent recovery optimization that increases operational complexity. SWE offers environmentally sustainable extraction with superior bioactivity profiles and zero chemical residues, positioning it as the most ecologically favorable option for large-scale applications. Nevertheless, thermal optimization remains critical—balancing maximum polyphenol recovery against degradation of heat-labile compounds such as flavonols and stilbenes demands precise parameter control tailored to each specific biomass type. MPR provides cost-effective purification with reusable matrices and easily recoverable desorption solvents, but sacrifices synergistic compound interactions by eliminating minor polyphenols from crude extracts, potentially reducing the holistic bioactivity benefits characterizing multi-component natural extracts. Consequently, extraction methodology should be selected based on specific application: DES for diverse polyphenol profiles, SWE for sustainable large-scale production, and MPR for standardized single-compound isolation.

The hypothesis that food waste-derived polyphenols exhibit high versatility as ligands with promising inhibitory effects is supported by comprehensive drug-likeness evaluation. Analysis of 169 selected polyphenols revealed that 52.1 % exhibited zero Lipinski rule violations, while 47.9 % demonstrated one to three violations, indicating generally favorable drug-likeness. Polyphenols function as non-selective, promiscuous ligands targeting multiple enzyme families critical to pathogenic pathways, with key inhibitory targets including aldose reductase, xanthine oxidase, cyclooxygenase-2, phosphodiesterase, adenosine deaminase, and lipoxygenase. This broad inhibition spectrum provides therapeutic potential against cancers, inflammatory diseases, diabetes and obesity, cardiovascular diseases, and mental and neurological disorders—directly addressing the central hypothesis of multi-target disease mitigation.

Polyphenol consumption via dietary sources provides therapeutic benefits with minimal adverse effects at physiological doses, whereas purified polyphenol supplements present distinct safety concerns requiring stratified recommendations. Moderate-dose supplementation shows promise for managing gastrointestinal disorders such as IBS and IBDs through selective prebiotic effects—promoting beneficial bacteria while inhibiting pathogenic species—and subsequent microbial generation of bioactive metabolites. Conversely, high-dose purified supplements carry significant risks including inhibition of digestive enzymes causing nutrient malabsorption, iron sequestration precipitating anemia in vulnerable populations, cytochrome P450 enzyme inhibition creating dangerous drug interactions with anticoagulants and statins, and dysbiosis through excessive antimicrobial activity. Furthermore, estrogenic activity from isoflavonoids and thyroid peroxidase inhibition raise concerns for hypothyroidism development in iodine-deficient individuals. Low bioavailability of dietary polyphenols—only 5–10 % absorbed in the small intestine, with 90–95 % reaching the large intestine where extensive microbial metabolism generates bioactive metabolites—combined with substantial inter-individual variability influenced by gut microbiota composition, intestinal enzyme activity, genetic polymorphisms, and dietary matrix effects, underscores the importance of dietary diversity. The hormesis principle indicates that consuming diverse polyphenols substantially increases the total dose required to reach cytotoxic thresholds compared to high-dose supplementation of single compounds.

Food and food waste biomasses represent underutilized reservoirs and can be processed into food-grade, high-concentration polyphenol dietary supplements through sequential extraction protocols combining SWE and DES technologies, followed by MPR purification. A tiered approach is recommended: (1) for disease prevention, emphasize whole food sources at physiological doses; (2) for specific disease management of IBS, IBDs, and mild-to-moderate colitis, moderate-dose supplements derived from food waste valorization may provide therapeutic benefit under healthcare provider supervision; and (3) for vulnerable populations, dietary polyphenol intake should be assessed individually, with high-dose supplementation contraindicated unless clinically monitored. Quality control standards must address adulteration, heavy metal contamination, pesticide residues, and accurate labeling. While polyphenols demonstrate versatile inhibitory potential against pathogenic proteins and enzymes, translating molecular evidence into clinical efficacy requires rigorous in vivo validation, mechanistic elucidation of microbiota-dependent effects, standardization of extraction and isolation methods, and development of evidence-based supplementation guidelines. The integration of polyphenol-rich food waste into sustainable biorefinery frameworks offers significant potential for personalized nutraceutical development while advancing circular economy principles in the food industry.

## Data sharing

The manuscript is a review article and does not report on original data. All data and information presented are derived from previously published studies and are fully cited within the text.

## CRediT authorship contribution statement

**Malthe Fredsgaard:** Writing – review & editing, Writing – original draft, Visualization, Formal analysis, Data curation, Conceptualization. **Andre Fussy:** Writing – review & editing, Writing – original draft, Visualization, Formal analysis, Data curation, Conceptualization. **Gowri Købke Nybo:** Writing – original draft. **Jutta Papenbrock:** Writing – review & editing, Writing – original draft, Supervision. **Laura Sini Sofia Hulkko:** Writing – original draft. **Mina Dadjoo:** Writing – original draft. **Tanmay Chaturvedi:** Writing – original draft. **Mette Hedegaard Thomsen:** Writing – review & editing, Writing – original draft, Supervision.

## Declaration of generative AI and AI-assisted technologies in the writing process

No generative AI or AI-assisted technologies were used in the writing process of this manuscript.

## Funding

This study was initiated within the AQUACOMBINE research project funded by the European Union's Horizon 2020 research and innovation programme under Grant Agreement No. 862834. Any results of this project reflect only this consortium's view, and the European Commission is not responsible for any use that may be made of the information it contains.

## Declaration of competing interest

The authors declare that they have no known competing financial interests or personal relationships that could have appeared to influence the work reported in this paper.

## Data Availability

Data will be made available on request.

## Appendix A Supplementary data

Supplementary material: Table A.10 – Selected polyphenols in the review and their structural description and Table A.11 – Polyphenols and drug-likeness through number of Lipinski violations including rotatable bonds. Image 1
