# Supplementary material for: Polyphenols in food and food wastes: Extraction, isolation, and health applications
Source: Food Chem (Oxf). 2026 Jan 2;12:100351. doi: 10.1016/j.fochms.2025.100351 (PMC12810567; doi:10.1016/j.fochms.2025.100351)
Supplement: Supplementary file 1 — Supplementary material: Table A.10 – Selected polyphenols in the review and their structural description and Table A.11 – Polyphenols and drug-likeness through number of Lipinski violations including rotatable bonds. [file mmc1.docx]

# Appendix. Supplementary materials

Table A.10 – Selected polyphenols in the review and their structural description. Structural representations are found in Figure 3. H: Hydrogen. OH: Hydroxyl. O: Oxygen. COOH: Carboxyl group. Glu: Glucoside. Arab: Arabinoside. Furan: Furanoside. Gal: Galactoside. Xyl: Xyloside. Me: Methyl. Rut: Rutinoside. Gluc: Glucuronoside. Rha: Rhamnoside. Cou: p-coumaryl. Sop: Sophoroside. Tar: Tartaric acid. Qui: Quinic acid. Caf: Caffeic acid.

| **Polyphenols** | **IUPAC name** | **2** | **3** | **4** | **5** | **6** | **7** | **8** | **2’** | **3’** | **4’** | **5’** | **6’** |
| --- | --- | --- | --- | --- | --- | --- | --- | --- | --- | --- | --- | --- | --- |
| Flavonols | | | | | | | | | | | | | |
| Astragalin | 5,7-dihydroxy-2-(4-hydroxyphenyl)-3-[(2S,3R,4S,5S,6R)-3,4,5-trihydroxy-6-(hydroxymethyl)oxan-2-yl]oxychromen-4-one |  | OGlu | =O | H | H | H | OH | H | H | OH | H | H |
| Avicularin | 3-[(2S,3R,4R,5S)-3,4-dihydroxy-5-(hydroxymethyl)oxolan-2-yl]oxy-2-(3,4-dihydroxyphenyl)-5,7-dihydroxychromen-4-one |  | OArabFuran | =O | OH | H | OH | H | H | H | OH | OH | H |
| Fisetin | 2-(3,4-dihydroxyphenyl)-3,7-dihydroxychromen-4-one |  | OH | =O | H | H | OH | H | H | H | OH | OH | H |
| Galangin | 3,5,7-trihydroxy-2-phenylchromen-4-one |  | OH | =O | OH | H | OH | H | H | H | H | H | H |
| Hyperoside | 2-(3,4-dihydroxyphenyl)-5,7-dihydroxy-3-[(2S,3R,4S,5R,6R)-3,4,5-trihydroxy-6-(hydroxymethyl)oxan-2-yl]oxychromen-4-one |  | OGal | =O | OH | H | OH | H | H | H | OH | OH | H |
| Isoquercetrin | 3-[(2S,3R,4R,5R)-5-[(1R)-1,2-dihydroxyethyl]-3,4-dihydroxyoxolan-2-yl]oxy-2-(3,4-dihydroxyphenyl)-5,7-dihydroxychromen-4-one |  | OGlu | =O | OH | H | OH | H | H | H | OH | OH | H |
| Isorhamnetin | 3,5,7-trihydroxy-2-(4-hydroxy-3-methoxyphenyl)chromen-4-one |  | OH | =O | OH | H | OH | H | H | OMe | OH | H | H |
| Isorhamnetin-3-galactoside | 5,7-dihydroxy-2-(4-hydroxy-3-methoxyphenyl)-3-[3,4,5-trihydroxy-6-(hydroxymethyl)oxan-2-yl]oxychromen-4-one |  | OGal | =O | OH | H | OH | H | H | OMe | OH | H | H |
| Isorhamnetin-3-glucoside | 5,7-dihydroxy-2-(4-hydroxy-3-methoxyphenyl)-3-[(2S,3R,4S,5S,6R)-3,4,5-trihydroxy-6-(hydroxymethyl)oxan-2-yl]oxychromen-4-one |  | OGlu | =O | OH | H | OH | H | H | OMe | OH | H | H |
| Isorhamnetin-3-xyloside | 5,7-dihydroxy-2-(4-hydroxy-3-methoxyphenyl)-3-[(2S,5R)-3,4,5-trihydroxyoxan-2-yl]oxychromen-4-one |  | OXyl | =O | OH | H | OH | H | H | OMe | OH | H | H |
| Kaempferol | 3,5,7-trihydroxy-2-(4-hydroxyphenyl)chromen-4-one |  | OH | =O | OH | H | OH | H | H | H | OH | H | H |
| Kaempferol-3-glucuronide | (2S,3S,4S,5R)-6-[5,7-dihydroxy-2-(4-hydroxyphenyl)-4-oxochromen-3-yl]oxy-3,4,5-trihydroxyoxane-2-carboxylic acid |  | OH | =O | OH | H | OGluc | H | H | H | OH | H | H |
| Morin | 2-(2,4-dihydroxyphenyl)-3,5,7-trihydroxychromen-4-one |  | OH | =O | OH | H | OH | H | OH | H | OH | H | H |
| Myricetin | 3,5,7-trihydroxy-2-(3,4,5-trihydroxyphenyl)chromen-4-one |  | OH | =O | OH | H | OH | H | H | OH | OH | OH | H |
| Myricetin-3-galactoside | 5,7-dihydroxy-3-[(2S,3R,4S,5R,6R)-3,4,5-trihydroxy-6-(hydroxymethyl)oxan-2-yl]oxy-2-(3,4,5-trihydroxyphenyl)chromen-4-one |  | OGal | =O | OH | H | OH | H | H | OH | OH | OH | H |
| Myricetin-3-xyloside | 5,7-dihydroxy-3-[(2S, 5R)-3,4,5-trihydroxyoxan-2-yl]oxy-2-(3,4,5-trihydroxyphenyl)chromen-4-one |  | OXyl | =O | OH | H | OH | H | H | OH | OH | OH | H |
| Myricitrin | 5,7-dihydroxy-3-[(2S,3R,4R,5R,6S)-3,4,5-trihydroxy-6-methyloxan-2-yl]oxy-2-(3,4,5-trihydroxyphenyl)chromen-4-one |  | OGlu | =O | OH | H | OH | H | H | OH | OH | OH | H |
| Narcissin | 5,7-dihydroxy-2-(4-hydroxy-3-methoxyphenyl)-3-[(2S,3R,4S,5S,6R)-3,4,5-trihydroxy-6-[[(2R,3R,4R,5R,6S)-3,4,5-trihydroxy-6-methyloxan-2-yl]oxymethyl]oxan-2-yl]oxychromen-4-one |  | ORut | =O | OH | H | OH | H | H | OMe | OH | H | H |
| Nicotiflorin | 5,7-dihydroxy-2-(4-hydroxyphenyl)-3-[(2S,3R,4S,5S,6R)-3,4,5-trihydroxy-6-[[(2R,3R,4R,5R,6S)-3,4,5-trihydroxy-6-methyloxan-2-yl]oxymethyl]oxan-2-yl]oxychromen-4-one |  | ORut | =O | OH | H | OH | H | H | H | OH | H | H |
| Patuletin | 2-(3,4-dihydroxyphenyl)-3,5,7-trihydroxy-6-methoxychromen-4-one |  | OH | =O | OH | OMe | OH | H | H | H | OH | OH | H |
| Patuletin-7-glucoside | 2-(3,4-dihydroxyphenyl)-3,5-dihydroxy-6-methoxy-7-[3,4,5-trihydroxy-6-(hydroxymethyl)oxan-2-yl]oxychromen-4-one |  | OH | =O | OH | OMe | OGlu | H | H | OH | OH | H | H |
| Quercetagetin | 2-(3,4-dihydroxyphenyl)-3,5,6,7-tetrahydroxychromen-4-one |  | OH | =O | OH | OH | OH | H | H | OH | OH | H | H |
| Quercetin | 2-(3,4-dihydroxyphenyl)-3,5,7-trihydroxychromen-4-one |  | OH | =O | OH | H | OH | H | H | H | OH | OH | H |
| Quercetin-3-arabinoside | 3-[(2R,3R,4R,5S)-3,4-dihydroxy-5-(hydroxymethyl)oxolan-2-yl]oxy-2-(3,4-dihydroxyphenyl)-5,7-dihydroxychromen-4-one |  | OArab | =O | OH | H | OH | H | H | H | OH | OH | H |
| Quercetin-3-methyl ether | 2-(3,4-dihydroxyphenyl)-5,7-dihydroxy-3-methoxychromen-4-one |  | OMe | =O | OH | H | OH | H | H | H | OH | OH | H |
| Quercetin-3-xyloside | 3-[(2S,3R,4R,5R)-3,4-dihydroxy-5-(hydroxymethyl)oxolan-2-yl]oxy-2-(3,4-dihydroxyphenyl)-5,7-dihydroxychromen-4-one |  | OXyl | =O | OH | H | OH | H | H | H | OH | OH | H |
| Quercitrin | 2-(3,4-dihydroxyphenyl)-5,7-dihydroxy-3-[(2S,3R,4R,5R,6S)-3,4,5-trihydroxy-6-methyloxan-2-yl]oxychromen-4-one |  | ORha | =O | OH | H | OH | H | H | OH | OH | H | H |
| Querciturone | (2S,3S,4S,5R,6S)-6-[2-(3,4-dihydroxyphenyl)-5,7-dihydroxy-4-oxochromen-3-yl]oxy-3,4,5-trihydroxyoxane-2-carboxylic acid |  | OGluc | =O | OH | H | OH | H | H | OH | OH | H | H |
| Rutin | 2-(3,4-dihydroxyphenyl)-5,7-dihydroxy-3-[(2S,3R,4S,5S,6R)-3,4,5-trihydroxy-6-[[(2R,3R,4R,5R,6S)-3,4,5-trihydroxy-6-methyloxan-2-yl]oxymethyl]oxan-2-yl]oxychromen-4-one |  | ORut | =O | OH | H | OH | H | H | OH | OH | H | H |
| Spinacetin | 3,5,7-trihydroxy-2-(4-hydroxy-3-methoxyphenyl)-6-methoxychromen-4-one |  | OH | =O | OH | OMe | OH | H | H | OMe | OH | H | H |
| Taxifolin | (2R,3R)-2-(3,4-dihydroxyphenyl)-3,5,7-trihydroxy-2,3-dihydrochromen-4-one |  | OH | =O | OH | H | OH | H | H | H | OH | OH | H |
| Tiliroside | [(2R,3S,4S,5R,6S)-6-[5,7-dihydroxy-2-(4-hydroxyphenyl)-4-oxochromen-3-yl]oxy-3,4,5-trihydroxyoxan-2-yl]methyl (E)-3-(4-hydroxyphenyl)prop-2-enoate |  | OGluOCou | =O | OH | H | OH | H | H | H | OH | H | H |
| Trifolin | 5,7-dihydroxy-2-(4-hydroxyphenyl)-3-[(2S,3R,4S,5R,6R)-3,4,5-trihydroxy-6-(hydroxymethyl)oxan-2-yl]oxychromen-4-one |  | OGal | =O | OH | H | OH | H | H | H | OH | H | H |
| 5-Deoxykaempferol | 3,7-dihydroxy-2-(4-hydroxyphenyl)chromen-4-one |  | OH | =O | H | H | OH | H | H | H | OH | H | H |
| 6-Hydroxykaempferol-3-glucoside | 5,6,7-trihydroxy-2-(4-hydroxyphenyl)-3-[(2S,3R,4S,5S,6R)-3,4,5-trihydroxy-6-(hydroxymethyl)oxan-2-yl]oxychromen-4-one |  | OGlu | =O | OH | OH | OH | H | H | H | OH | H | H |
| Flavones | | | | | | | | | | | | | |
| Acacetin | 5,7-dihydroxy-2-(4-methoxyphenyl)chromen-4-one |  | H | =O | OH | H | OH | H | H | H | OMe | H | H |
| Apigenin | 5,7-dihydroxy-2-(4-hydroxyphenyl)chromen-4-one |  | H | =O | OH | H | OH | H | H | H | OH | H | H |
| Apigenin-7-glucuronide | (2S,3S,4S,5R,6S)-3,4,5-trihydroxy-6-[5-hydroxy-2-(4-hydroxyphenyl)-4-oxochromen-7-yl]oxyoxane-2-carboxylic acid |  | H | =O | OH | H | OGluc | H | H | H | OH | H | H |
| Apigetrin | 5-hydroxy-2-(4-hydroxyphenyl)-7-[(2S,3R,4S,5S,6R)-3,4,5-trihydroxy-6-(hydroxymethyl)oxan-2-yl]oxychromen-4-one |  | H | =O | OH | H | OGlu | H | H | H | OH | H | H |
| Baicalein | 5,6,7-trihydroxy-2-phenylchromen-4-one |  | H | =O | OH | OH | OH | H | H | H | H | H | H |
| Chrysin | 5,7-dihydroxy-2-phenylchromen-4-one |  | H | =O | OH | H | OH | H | H | H | H | H | H |
| Cirsimaritin | 5-hydroxy-2-(4-hydroxyphenyl)-6,7-dimethoxychromen-4-one |  | H | =O | OH | OMe | OMe | H | H | H | OH | H | H |
| Cyranoside | 2-(3,4-dihydroxyphenyl)-5-hydroxy-7-[(2S,3R,4S,5S,6R)-3,4,5-trihydroxy-6-(hydroxymethyl)oxan-2-yl]oxychromen-4-one |  | H | =O | OH | H | OGlu | H | H | H | OH | OH | H |
| Diosmetin | 5,7-dihydroxy-2-(3-hydroxy-4-methoxyphenyl)chromen-4-one |  | H | =O | OH | H | OH | H | H | H | OMe | OH | H |
| Diosmin | 5-hydroxy-2-(3-hydroxy-4-methoxyphenyl)-7-[(2S,3R,4S,5S,6R)-3,4,5-trihydroxy-6-[[(2R,3R,4R,5R,6S)-3,4,5-trihydroxy-6-methyloxan-2-yl]oxymethyl]oxan-2-yl]oxychromen-4-one |  | H | =O | OH | H | ORha | H | H | OH | OMe | H | H |
| Fastigenin | 5-hydroxy-2-(4-hydroxy-3-methoxyphenyl)-6,7-dimethoxychromen-4-one |  | H | =O | OH | OMe | OMe | H | H | OMe | OH | H | H |
| Genkwanin | 5-hydroxy-2-(4-hydroxyphenyl)-7-methoxychromen-4-one |  | H | =O | OH | H | OMe | H | H | H | OH | H | H |
| Isoorientin | 2-(3,4-dihydroxyphenyl)-5,7-dihydroxy-6-[(2S,3R,4R,5S,6R)-3,4,5-trihydroxy-6-(hydroxymethyl)oxan-2-yl]chromen-4-one |  | H | =O | OH | OGlu | OH | H | H | OH | OH | H | H |
| Isovitexin | 5,7-dihydroxy-2-(4-hydroxyphenyl)-6-[(2S,3R,4R,5S,6R)-3,4,5-trihydroxy-6-(hydroxymethyl)oxan-2-yl]chromen-4-one |  | H | =O | OH | OGlu | OH | H | H | H | OH | H | H |
| Luteolin | 2-(3,4-dihydroxyphenyl)-5,7-dihydroxychromen-4-one |  | H | =O | OH | H | OH | H | H | OH | OH | O | H |
| Luteolin-7-glucuronide | (2S,3S,4S,5R,6S)-6-[2-(3,4-dihydroxyphenyl)-5-hydroxy-4-oxochromen-7-yl]oxy-3,4,5-trihydroxyoxane-2-carboxylic acid |  | H | =O | OH | H | OGluc | H | O | OH | OH | H | H |
| Orientin | 2-(3,4-dihydroxyphenyl)-5,7-dihydroxy-8-[(2S,3R,4R,5S,6R)-3,4,5-trihydroxy-6-(hydroxymethyl)oxan-2-yl]chromen-4-one |  | H | =O | OH | H | OH | OGlu | H | H | OH | OH | H |
| Pebrellin | 2-(3,4-dimethoxyphenyl)-5,6-dihydroxy-7,8-dimethoxychromen-4-one |  | H | =O | OH | OH | OMe | OMe | H | OMe | OMe | H | H |
| Tangeretin | 5,6,7,8-tetramethoxy-2-(4-methoxyphenyl)chromen-4-one |  | H | =O | OMe | OMe | OMe | OMe | H | H | OMe | H | H |
| Tricetin | 5,7-dihydroxy-2-(3,4,5-trihydroxyphenyl)chromen-4-one |  | H | =O | OH | H | OH | H | H | OH | OH | OH | H |
| Tricin | 5,7-dihydroxy-2-(4-hydroxy-3,5-dimethoxyphenyl)chromen-4-one |  | H | =O | OH | H | OH | H | H | OMe | OH | OMe | H |
| Vicenin 2 | 5,7-dihydroxy-2-(4-hydroxyphenyl)-6,8-bis[(2S,3R,4R,5S,6R)-3,4,5-trihydroxy-6-(hydroxymethyl)oxan-2-yl]chromen-4-one |  | H | =O | OH | OGlu | OH | OGlu | H | H | OH | H | H |
| Vitexin | 5,7-dihydroxy-2-(4-hydroxyphenyl)-8-[(2S,3R,4R,5S,6R)-3,4,5-trihydroxy-6-(hydroxymethyl)oxan-2-yl]chromen-4-one |  | H | =O | OH | H | OH | OGlu | H | H | OH | H | H |
| Isoflavones | | | | | | | | | | | | | |
| Daidzein | 7-hydroxy-3-(4-hydroxyphenyl)chromen-4-one | H |  | =O | H | H | OH | H | H | H | OH | H | H |
| Demethyltexasin | 6,7-dihydroxy-3-(4-hydroxyphenyl)chromen-4-one | H |  | =O | H | OH | OH | H | H | H | OH | H | H |
| Genistein | 5,7-dihydroxy-3-(4-hydroxyphenyl)chromen-4-one | H |  | =O | OH | H | OH | H | H | H | OH | H | H |
| 3'-Hydroxydaidzein | 3-(3,4-dihydroxyphenyl)-7-hydroxychromen-4-one | H |  | =O | H | H | OH | H | H | OH | OH | H | H |
| Flavanols | | | | | | | | | | | | | |
| Catechin | (2R,3S)-2-(3,4-dihydroxyphenyl)-3,4-dihydro-2H-chromene-3,5,7-triol |  | OH | H | OH | H | OH | H | H | H | OH | OH | H |
| Epicatechin | (2R,3R)-2-(3,4-dihydroxyphenyl)-3,4-dihydro-2H-chromene-3,5,7-triol |  | OH | H | OH | H | OH | H | H | H | OH | OH | H |
| Epigallocatechin | (2R,3R)-2-(3,4,5-trihydroxyphenyl)-3,4-dihydro-2H-chromene-3,5,7-triol |  | OH | H | OH | H | OH | H | H | OH | OH | OH | H |
| Epigallocatechin gallate | [(2R,3R)-5,7-dihydroxy-2-(3,4,5-trihydroxyphenyl)-3,4-dihydro-2H-chromen-3-yl] 3,4,5-trihydroxybenzoate |  | OGal | H | OH | H | OH | H | H | OH | OH | OH | H |
| Gallocatechin | (2R,3S)-2-(3,4,5-trihydroxyphenyl)-3,4-dihydro-2H-chromene-3,5,7-triol |  | OH | H | OH | H | OH | H | H | OH | OH | OH | H |
| Isoflavanes | | | | | | | | | | | | | |
| Equol | (3S)-3-(4-hydroxyphenyl)-3,4-dihydro-2H-chromen-7-ol | H |  | H | H | H | OH | H | H | H | OH | H | H |
| Flavanones | | | | | | | | | | | | | |
| Dihydrokaempferol-3-glucoside | (2R,3R)-5,7-dihydroxy-2-(4-hydroxyphenyl)-3-[(2S,3R,4S,5S,6R)-3,4,5-trihydroxy-6-(hydroxymethyl)oxan-2-yl]oxy-2,3-dihydrochromen-4-one |  | OGlu | =O | OH | H | OH | H | H | H | OH | H | H |
| Eriocitrin | (2S)-2-(3,4-dihydroxyphenyl)-5-hydroxy-7-[(2S,3R,4S,5S,6R)-3,4,5-trihydroxy-6-[[(2R,3R,4R,5R,6S)-3,4,5-trihydroxy-6-methyloxan-2-yl]oxymethyl]oxan-2-yl]oxy-2,3-dihydrochromen-4-one |  | H | =O | OH | H | ORut | H | H | OH | OH | H | H |
| Eriodictyol | (2S)-2-(3,4-dihydroxyphenyl)-5,7-dihydroxy-2,3-dihydrochromen-4-one |  | H | H | OH | H | OH | H | H | H | OH | OH | H |
| Hesperidin | (2S)-5-hydroxy-2-(3-hydroxy-4-methoxyphenyl)-7-[(2S,3R,4S,5S,6R)-3,4,5-trihydroxy-6-[[(2R,3R,4R,5R,6S)-3,4,5-trihydroxy-6-methyloxan-2-yl]oxymethyl]oxan-2-yl]oxy-2,3-dihydrochromen-4-one |  | H | =O | OH | H | ORut | H | H | H | OMe | OH | H |
| Melitidin | 5-[[(2R,3S,4S,5R,6S)-3,4-Dihydroxy-6-[[(2S)-5-hydroxy-2-(4-hydroxyphenyl)-4-oxo-2,3-dihydrochromen-7-yl]oxy]-5-[(2S,3R,4R,5R,6S)-3,4,5-trihydroxy-6-methyloxan-2-yl]oxyoxan-2-yl]methoxy]-3-hydroxy-3-methyl-5-oxopentanoic acid |  | H | =O | OH | H | ORhaGlu polyether polyol | H | H | H | OH | H | H |
| Naringin | (2S)-7-[(2S,3R,4S,5S,6R)-4,5-dihydroxy-6-(hydroxymethyl)-3-[(2S,3R,4R,5R,6S)-3,4,5-trihydroxy-6-methyloxan-2-yl]oxyoxan-2-yl]oxy-5-hydroxy-2-(4-hydroxyphenyl)-2,3-dihydrochromen-4-one |  | H | =O | OH | H | ORhaGlu | H | H | H | OH | H | H |
| Naringenin | (2S)-5,7-dihydroxy-2-(4-hydroxyphenyl)-2,3-dihydrochromen-4-one |  | H | =O | OH | H | OH | H | H | H | OH | H | H |
| Narirutin | (2S)-5-hydroxy-2-(4-hydroxyphenyl)-7-[(2S,3R,4S,5S,6R)-3,4,5-trihydroxy-6-[[(2R,3R,4R,5R,6S)-3,4,5-trihydroxy-6-methyloxan-2-yl]oxymethyl]oxan-2-yl]oxy-2,3-dihydrochromen-4-one |  | H | =O | OH | H | ORut | H | H | H | OH | H | H |
| Neohesperidin | (2S)-7-[(2S,3R,4S,5S,6R)-4,5-dihydroxy-6-(hydroxymethyl)-3-[(2S,3R,4R,5R,6S)-3,4,5-trihydroxy-6-methyloxan-2-yl]oxyoxan-2-yl]oxy-5-hydroxy-2-(3-hydroxy-4-methoxyphenyl)-2,3-dihydrochromen-4-one |  | H | =O | OH | H | ORut | H | H | H | OMe | OH | H |
| Pinocembrin | (2S)-5,7-dihydroxy-2-phenyl-2,3-dihydrochromen-4-one |  | H | =O | OH | H | OH | H | H | H | H | H | H |
| Anthocyanins | | | | | | | | | | | | | |
| Antirrhinin (synonym of Keracyanin) | (2S,3R,4S,5S,6R)-2-[2-(3,4-dihydroxyphenyl)-5,7-dihydroxychromenylium-3-yl]oxy-6-[[(2R,3R,4R,5R,6S)-3,4,5-trihydroxy-6-methyloxan-2-yl]oxymethyl]oxane-3,4,5-triol chloride |  | ORut | H | OH | H | OH | H | H | OH | OH | H | H |
| Apigenidin | 2-(4-hydroxyphenyl)chromenylium-5,7-diol;chloride |  | H | H | OH | H | OH | H | H | H | OH | H | H |
| Callistephin | (2S,3R,4S,5S,6R)-2-[5,7-dihydroxy-2-(4-hydroxyphenyl)chromenylium-3-yl]oxy-6-(hydroxymethyl)oxane-3,4,5-triol;chloride |  | OGlu | H | OH | H | OH | H | H | H | OH | H | H |
| Cyanidin | 2-(3,4-dihydroxyphenyl)chromenylium-3,5,7-triol |  | OH | H | OH | H | OH | H | H | OH | OH | H | H |
| Cyanidin-3-sophoroside | (2S,3R,5S)-2-[(2S,5S)-2-[2-(3,4-dihydroxyphenyl)-5,7-dihydroxychromenylium-3-yl]oxy-4,5-dihydroxy-6-(hydroxymethyl)oxan-3-yl]oxy-6-(hydroxymethyl)oxane-3,4,5-triol |  | OSop | H | OH | H | OH | H | H | OH | OH | H | H |
| Cyanin | (2S,3R,4S,5S,6R)-2-[2-(3,4-dihydroxyphenyl)-7-hydroxy-3-[(2S,3R,4S,5S,6R)-3,4,5-trihydroxy-6-(hydroxymethyl)oxan-2-yl]oxychromenylium-5-yl]oxy-6-(hydroxymethyl)oxane-3,4,5-triol |  | OGlu | H | OGlu | H | OH | H | H | H | OH | OH | H |
| Delphinidin | 2-(3,4,5-trihydroxyphenyl)chromenylium-3,5,7-triol;chloride |  | OH | H | OH | H | OH | H | H | OH | OH | OH | H |
| Delphinidin-3,5-diglucoside | (2R,3S,4S,5R,6S)-2-(hydroxymethyl)-6-[7-hydroxy-3-[(2S,3R,4S,5S,6R)-3,4,5-trihydroxy-6-(hydroxymethyl)oxan-2-yl]oxy-2-(3,4,5-trihydroxyphenyl)chromenylium-5-yl]oxyoxane-3,4,5-triol |  | OGlu | H | OGlu | H | =O | H | H | OH | OH | OH | H |
| Keracyanin (synonym of Antirrhinin) | (2R,3R,4R,5R,6S)-2-[[(2R,3S,4S,5R,6S)-6-[2-(3,4-dihydroxyphenyl)-5,7-dihydroxychromenylium-3-yl]oxy-3,4,5-trihydroxyoxan-2-yl]methoxy]-6-methyloxane-3,4,5-triol;chloride |  | ORut | H | OH | H | OH | H | H | H | OH | OH | H |
| Kuromanin | (2S,3R,4S,5S,6R)-2-[2-(3,4-dihydroxyphenyl)-5,7-dihydroxychromenylium-3-yl]oxy-6-(hydroxymethyl)oxane-3,4,5-triol;chloride |  | OGlu | H | OH | H | OH | H | H | H | OH | OH | H |
| Malvin | (2S,3R,4S,5S,6R)-2-[7-hydroxy-2-(4-hydroxy-3,5-dimethoxyphenyl)-3-[(2S,3R,4S,5S,6R)-3,4,5-trihydroxy-6-(hydroxymethyl)oxan-2-yl]oxychromenylium-5-yl]oxy-6-(hydroxymethyl)oxane-3,4,5-triol;chloride |  | OGlu | H | OGlu | H | OH | H | H | OMe | OH | OMe | H |
| Myrtillin | (2S,3R,4S,5S,6R)-2-[5,7-dihydroxy-2-(3,4,5-trihydroxyphenyl)chromenylium-3-yl]oxy-6-(hydroxymethyl)oxane-3,4,5-triol;chloride |  | OGlu | H | OH | H | =O | H | H | OH | OH | OH | H |
| Pelargonin | (2S,3R,4S,5S,6R)-2-[7-hydroxy-2-(4-hydroxyphenyl)-3-[(2S,3R,4S,5S,6R)-3,4,5-trihydroxy-6-(hydroxymethyl)oxan-2-yl]oxychromenylium-5-yl]oxy-6-(hydroxymethyl)oxane-3,4,5-triol |  | OGlu | H | OGlu | H | OH | H | H | H | OH | H | H |
| Pelargonidin | 2-(4-hydroxyphenyl)chromenylium-3,5,7-triol |  | OH | H | OH | H | OH | H | H | H | OH | H | H |
| Peonidin-3-glucoside | (2S,3R,4S,5S,6R)-2-[5,7-dihydroxy-2-(4-hydroxy-3-methoxyphenyl)chromenylium-3-yl]oxy-6-(hydroxymethyl)oxane-3,4,5-triol |  | OGlu | H | OH | H | OH | H | H | H | OH | OMe | H |
| Peonin | (2S,4S,5S)-2-[7-hydroxy-2-(4-hydroxy-3-methoxyphenyl)-3-[(2S,5S)-3,4,5-trihydroxy-6-(hydroxymethyl)oxan-2-yl]oxychromenylium-5-yl]oxy-6-(hydroxymethyl)oxane-3,4,5-triol |  | OGlu | H | OGlu | H | OH | H | H | H | OH | OMe | H |
| Peonidin-3-(p-coumaroyl)-rutinoside-5-glucoside | [(2S,3R,4S,5R,6R)-4,5-dihydroxy-2-methyl-6-[[(2R,3S,4S,5R,6S)-3,4,5-trihydroxy-6-[7-hydroxy-2-(4-hydroxy-3-methoxyphenyl)-5-[(2S,3R,4S,5S,6R)-3,4,5-trihydroxy-6-(hydroxymethyl)oxan-2-yl]oxychromenylium-3-yl]oxyoxan-2-yl]methoxy]oxan-3-yl] (E)-3-(4-hydroxyphenyl)prop-2-enoate |  | ORutCou | H | OGlu | H | OH | H | H | H | OH | OMe | H |
| Petunin | (2S,3R,4S,5S,6R)-2-[2-(3,4-dihydroxy-5-methoxyphenyl)-7-hydroxy-3-[(2S,3R,4S,5S,6R)-3,4,5-trihydroxy-6-(hydroxymethyl)oxan-2-yl]oxychromenylium-5-yl]oxy-6-(hydroxymethyl)oxane-3,4,5-triol;chloride |  | OGlu | H | OGlu | H | OH | H | H | OMe | OH | OH | H |
| Petunidin-3-glucoside | (2S,3R,4S,5S,6R)-2-[2-(3,4-dihydroxy-5-methoxyphenyl)-5,7-dihydroxychromenylium-3-yl]oxy-6-(hydroxymethyl)oxane-3,4,5-triol |  | OGlu | H | OH | H | OH | H | H | OMe | OH | OH |  |
| Chalcones | | | | | | | | | | | |  |  |
| Polyphenol | IUPAC name | 2 | 3 | 4 | 5 | 6 | 2’ | 3’ | 4’ | 5’ | 6’ |  |  |
| Phloretin | 3-(4-hydroxyphenyl)-1-(2,4,6-trihydroxyphenyl)propan-1-one | H | H | OH | H | H | OH | H | OH | H | OH |  |  |
| Phloridzin | 1-[2,4-dihydroxy-6-[(2S,3R,4S,5S,6R)-3,4,5-trihydroxy-6-(hydroxymethyl)oxan-2-yl]oxyphenyl]-3-(4-hydroxyphenyl)propan-1-one | H | H | OH | H | H | OH | H | OH | H | OGlu |  |  |
| Hydroxybenzoic acids | | | | | | | | |  |  |  |  |  |
| Polyphenol | IUPAC name | 2 | 3 | 4 | 5 | 6 | 1’ | 2’ |  |  |  |  |  |
| Benzoic acid | benzoic acid | H | H | H | H | H | OH | =O |  |  |  |  |  |
| Digallic acid | 3,4-dihydroxy-5-(3,4,5-trihydroxybenzoyl)oxybenzoic acid | H | OH | OH | OH | H | OGal | =O |  |  |  |  |  |
| Gallic acid | 3,4,5-trihydroxybenzoic acid | H | OH | OH | OH | H | OH | =O |  |  |  |  |  |
| Gentisic acid | 2,5-dihydroxybenzoic acid | H | OH | H | H | OH | OG | =O |  |  |  |  |  |
| Isovanillic acid | 3-hydroxy-4-methoxybenzoic acid | H | H | OMe | Oh | H | OH | =O |  |  |  |  |  |
| Methyl gallate | methyl 3,4,5-trihydroxybenzoate | H | OH | OH | OH | H | OMe | =O |  |  |  |  |  |
| Monogalloyl glucose | [(2S,3R,4S,5S,6R)-3,4,5-Trihydroxy-6-(hydroxymethyl)oxan-2-yl]3,4,5-trihydroxybenzoate | H | OH | OH | OH | H | OGlu | =O |  |  |  |  |  |
| Phthalic acid | Benzene-1,2-dicarboxylic acid | H | H | H | H | COOH | OH | =O |  |  |  |  |  |
| *p*-Hydroxybenzoic acid | 4-hydroxybenzoic acid | H | H | OH | H | H | OH | =O |  |  |  |  |  |
| Protocatechuic acid | 3,4-dihydroxybenzoic acid | H | H | OH | OH | H | OH | =O |  |  |  |  |  |
| Syringaldehyde | 4-hydroxy-3,5-dimethoxybenzaldehyde | H | OMe | OH | OMe | H | H | =O |  |  |  |  |  |
| Syringic acid | 4-hydroxy-3,5-dimethoxybenzoic acid | H | OMe | OH | OMe | H | OH | =O |  |  |  |  |  |
| Vanillic acid | 4-hydroxy-3-methoxybenzoic acid | H | H | OH | OMe | H | OH | =O |  |  |  |  |  |
| 4-Hydroxybenzaldehyde | 4-hydroxybenzaldehyde | H | H | OH | H | H | H | =O |  |  |  |  |  |
| Hydroxycinnamic acids | | | | | | | | |  |  |  |  |  |
| Caffeic acid | (E)-3-(3,4-dihydroxyphenyl)prop-2-enoic acid | H | OH | OH | H | H | OH | =O |  |  |  |  |  |
| Caftaric acid | (2R,3R)-2-[(E)-3-(3,4-dihydroxyphenyl)prop-2-enoyl]oxy-3-hydroxybutanedioic acid | H | H | OH | OH | H | OTar | =O |  |  |  |  |  |
| Chlorogenic acid | (1S,3R,4R,5R)-3-[(E)-3-(3,4-dihydroxyphenyl)prop-2-enoyl]oxy-1,4,5-trihydroxycyclohexane-1-carboxylic acid | H | OH | OH | H | H | OQui | =O |  |  |  |  |  |
| Cinnamic acid | (E)-3-phenylprop-2-enoic acid | H | H | H | H | H | OH | =O |  |  |  |  |  |
| Cryptochlorogenic acid | (3R,5R)-4-[(E)-3-(3,4-dihydroxyphenyl)prop-2-enoyl]oxy-1,3,5-trihydroxycyclohexane-1-carboxylic acid | H | OH | OH | H | H | OQui | =O |  |  |  |  |  |
| Ferulic acid | (E)-3-(4-hydroxy-3-methoxyphenyl)prop-2-enoic acid | H | OH | OMe | H | H | OH | =O |  |  |  |  |  |
| Linocaffein | (E)-3-[3-hydroxy-4-[(2S,3R,4S,5S,6R)-3,4,5-trihydroxy-6-(hydroxymethyl)oxan-2-yl]oxyphenyl]prop-2-enoic acid | H | H | OGlu | OH | H | OH | =O |  |  |  |  |  |
| Neochlorogenic acid | (1R,3R,4S,5R)-3-[(E)-3-(3,4-dihydroxyphenyl)prop-2-enoyl]oxy-1,4,5-trihydroxycyclohexane-1-carboxylic acid | H | OH | OH | H | H | OQui | =O |  |  |  |  |  |
| *p*-Coumaric acid | (E)-3-(4-hydroxyphenyl)prop-2-enoic acid | H | H | OH | H | H | OH | =O |  |  |  |  |  |
| *p*-Coumaroylquinic acid | (1S,3R,4R,5R)-1,3,4-trihydroxy-5-[(E)-3-(4-hydroxyphenyl)prop-2-enoyl]oxycyclohexane-1-carboxylic acid | H | H | OH | H | H | OQui | =O |  |  |  |  |  |
| Rosmarinic acid | (2R)-3-(3,4-dihydroxyphenyl)-2-[(E)-3-(3,4-dihydroxyphenyl)prop-2-enoyl]oxypropanoic acid | H | OH | OH | H | H | OCaf | =O |  |  |  |  |  |
| Sinapinic acid | (E)-3-(4-hydroxy-3,5-dimethoxyphenyl)prop-2-enoic acid | H | OMe | OH | OMe | H | OH | =O |  |  |  |  |  |
| 3,4-di-caffeoylquinic acid | (1S,3R,4R,5R)-3,4-bis[[(E)-3-(3,4-dihydroxyphenyl)prop-2-enoyl]oxy]-1,5-dihydroxycyclohexane-1-carboxylic acid | H | OH | OH | H | H | OQuiOCaf | =O |  |  |  |  |  |
| 3,5-di-caffeoylquinic acid | (3S,5S)-3,5-bis[[(E)-3-(3,4-dihydroxyphenyl)prop-2-enoyl]oxy]-1,4-dihydroxycyclohexane-1-carboxylic acid | H | OH | OH | H | H | OQuiOCaf | =O |  |  |  |  |  |

Table A.11 – Polyphenols and drug-likeness through number of Lipinski violations including rotatable bonds. LogP computed by XLogP 3.0 (Lipinski et al. 2012). HBA: Hydrogen bond acceptor. HBD: Hydrogen bond donor. ND: No data.

| **Polyphenols** | **M_mi_** | **HBD** | **HBA** | **LogP** | **Rotatable bonds** | **Lipinski violations** |
| --- | --- | --- | --- | --- | --- | --- |
| Flavonols | | | | | | |
| Astragalin | 448.10 | 7 | 11 | 0.7 | 4 | 2 |
| Avicularin | 434.08 | 7 | 11 | 1 | 4 | 2 |
| Fisetin | 286.05 | 4 | 6 | 2 | 1 | 0 |
| Galangin | 270.05 | 3 | 5 | 2.3 | 1 | 0 |
| Hyperoside | 464.10 | 8 | 12 | 0.4 | 4 | 2 |
| Isoquercetrin | 464.10 | 8 | 12 | -0.2 | 4 | 2 |
| Isorhamnetin | 316.06 | 4 | 7 | 1.9 | 2 | 0 |
| Isorhamnetin 3-galactoside | 478.11 | 7 | 12 | 0.7 | 5 | 2 |
| Isorhamnetin 3-glucoside | 478.11 | 7 | 12 | 0.7 | 5 | 2 |
| Isorhamnetin 3-xyloside | 448.10 | 6 | 11 | 0.8 | 4 | 2 |
| Kaempferol | 286.05 | 4 | 6 | 1.9 | 1 | 0 |
| Kaempferol 3-o-glucuronide | 462.08 | 7 | 12 | 1 | 4 | 2 |
| Morin | 302.04 | 5 | 7 | 1.5 | 1 | 0 |
| Myricetin | 318.04 | 6 | 8 | 1.2 | 1 | 1 |
| Myricetin 3-galactoside | 480.09 | 9 | 13 | 0 | 4 | 2 |
| Myricetin-3-xyloside | 450.08 | 8 | 12 | 0.1 | 3 | 2 |
| Myricitrin | 464.10 | 8 | 12 | 0.5 | 3 | 2 |
| Narcissin | 624.17 | 9 | 16 | -1 | 7 | 3 |
| Nicotiflorin | 594.16 | 9 | 15 | -0.9 | 6 | 3 |
| Patuletin | 332.05 | 5 | 8 | 2.1 | 2 | 0 |
| Patuletin 7-glucoside | 494.11 | 8 | 13 | 0.3 | 5 | 2 |
| Quercetagetin | 318.04 | 6 | 8 | 1.2 | 1 | 1 |
| Quercetin | 302.04 | 5 | 7 | 1.5 | 1 | 0 |
| Quercetin 3-arabinoside | 434.08 | 7 | 11 | 1 | 4 | 2 |
| Quercetin 3-methyl ether | 316.06 | 4 | 7 | 2.5 | 2 | 0 |
| Quercetin 3-xyloside | 434.08 | 7 | 11 | 1 | 4 | 2 |
| Quercitrin | 448.10 | 7 | 11 | 0.9 | 3 | 2 |
| Querciturone | 478.07 | 8 | 13 | 0.6 | 4 | 2 |
| Rutin | 610.15 | 10 | 16 | -1.3 | 6 | 3 |
| Spinacetin | 346.08 | 4 | 8 | 2.5 | 3 | 0 |
| Taxifolin | 304.06 | 5 | 7 | 1.5 | 1 | 0 |
| Tiliroside | 594.14 | 7 | 13 | 2.5 | 8 | 3 |
| Trifolin | 448.10 | 7 | 11 | 0.7 | 4 | 2 |
| 5-Deoxykaempferol | 270.05 | 3 | 5 | 2.7 | 1 | 0 |
| 6-Hydroxykaempferol 3-glucoside | 464.10 | 8 | 12 | 0.4 | 4 | 2 |
| Flavones | | | | | | |
| Acacetin | 284.07 | 2 | 5 | 2.1 | 2 | 0 |
| Apigenin | 270.05 | 3 | 5 | 1.7 | 1 | 0 |
| Apigenin 7-glucuronide | 446.08 | 6 | 11 | 0.2 | 4 | 2 |
| Apigetrin | 432.11 | 6 | 10 | -0.1 | 4 | 1 |
| Baicalein | 270.05 | 3 | 5 | 1.7 | 1 | 0 |
| Chrysin | 254.06 | 2 | 4 | 2.1 | 1 | 0 |
| Cirsimaritin | 314.08 | 2 | 6 | 2 | 3 | 0 |
| Cynaroside | 448.10 | 7 | 11 | 0.5 | 4 | 2 |
| Diosmetin | 300.06 | 3 | 6 | 1.7 | 2 | 0 |
| Diosmin | 608.17 | 8 | 15 | -0.8 | 7 | 3 |
| Fastigenin | 344.09 | 2 | 7 | 2.9 | 4 | 0 |
| Genkwanin | 284.07 | 2 | 5 | 2.1 | 2 | 0 |
| Isoorientin | 448.10 | 8 | 11 | -0.2 | 3 | 2 |
| Isovitexin | 432.11 | 7 | 10 | 0.2 | 3 | 1 |
| Luteolin | 286.05 | 4 | 6 | 1.4 | 1 | 0 |
| Luteolin 7-glucuronide | 462.08 | 7 | 12 | 0.8 | 4 | 2 |
| Orientin | 448.10 | 8 | 11 | -0.2 | 3 | 2 |
| Pebrellin | 374.10 | 2 | 8 | 2.9 | 5 | 0 |
| Tangeretin | 372.12 | 0 | 7 | 3 | 6 | 0 |
| Tricetin | 302.04 | 5 | 7 | 1 | 1 | 0 |
| Tricin | 330.07 | 3 | 7 | 1.7 | 3 | 0 |
| Vicenin-2 | 594.16 | 11 | 15 | -2.3 | 5 | 3 |
| Vitexin | 432.11 | 7 | 10 | 0.2 | 3 | 1 |
| Isoflavones | | | | | | |
| Daidzein | 254.06 | 2 | 4 | 2.5 | 1 | 0 |
| Demethyltexasin | 270.05 | 3 | 5 | 2.1 | 1 | 0 |
| Genistein | 270.05 | 3 | 5 | 2.7 | 1 | 0 |
| 3'-Hydroxydaidzein | 270.05 | 3 | 5 | 2.1 | 1 | 0 |
| Flavanols | | | | | | |
| Catechin | 290.08 | 5 | 6 | 0.4 | 1 | 0 |
| Epicatechin | 290.08 | 5 | 6 | 0.4 | 1 | 0 |
| Epigallocatechin | 306.07 | 6 | 7 | 0 | 1 | 0 |
| Epigallocatechin gallate | 458.08 | 8 | 11 | 1.2 | 4 | 2 |
| Eriodictyol | 288.06 | 4 | 6 | 2 | 1 | 0 |
| Gallocatechin | 306.07 | 6 | 7 | 0 | 1 | 1 |
| Isoflavans | | | | | | |
| Equol | 242.09 | 2 | 3 | 3 | 1 | 0 |
| Flavanones | | | | | | |
| Dihydrokaempferol 3-o-glucoside | 450.12 | 7 | 11 | 0.2 | 4 | 2 |
| Eriocitrin | 596.17 | 9 | 15 | -1.4 | 6 | 3 |
| Hesperidin | 610.19 | 8 | 15 | -1.1 | 7 | 3 |
| Naringin | 580.18 | 8 | 14 | -0.5 | 6 | 3 |
| Naringenin | 272.08 | 3 | 5 | 2.4 | 1 | 0 |
| Narirutin | 580.18 | 8 | 14 | -1.1 | 6 | 3 |
| Neohesperidin | 610.19 | 8 | 15 | -0.5 | 7 | 3 |
| Pinocembrin | 256.07 | 2 | 4 | 2.7 | 1 | 0 |
| Anthocyanins | | | | | | |
| Antirrhinin | 630.1 | 10 | 15 | ND | 6 | 3 |
| Apigenidin | 290.03 | 3 | 4 | ND | 1 | 0 |
| Callistephin | 468.08 | 7 | 10 | ND | 4 | 2 |
| Cyanidin | 287.06 | 5 | 5 | ND | 1 | 0 |
| Cyanidin 3-o-sophoroside | 611.16 | 11 | 15 | ND | 7 | 3 |
| Cyanin | 611.16 | 11 | 15 | ND | 7 | 3 |
| Delphinidin | 338.02 | 6 | 7 | ND | 1 | 1 |
| Delphinidin 3,5-o-diglucoside | 626.15 | 11 | 17 | -3.3 | 7 | 3 |
| Keracyanin | 630.14 | 10 | 15 | ND | 6 | 3 |
| Kuromanin | 484.08 | 8 | 11 | ND | 4 | 2 |
| Malvin | 690.16 | 10 | 17 | ND | 9 | 3 |
| Pelargonin | 595.17 | 10 | 14 | NA | 7 | 3 |
| Pelargonidin | 271.06 | 4 | 4 | NA | 1 | 0 |
| Peonidin 3-o-glucoside | 463.12 | 7 | 10 | NA | 5 | 1 |
| Peonin | 625.18 | 10 | 15 | NA | 8 | 3 |
| Chalcones | | | | | | |
| Phloretin | 274.08 | 4 | 5 | 2.6 | 4 | 0 |
| Phloridzin | 436.14 | 7 | 10 | 1.2 | 7 | 1 |
| Hydroxybenzoic acids | | | | | | |
| Benzoic acid | 122.04 | 1 | 2 | 1.9 | 1 | 0 |
| Digallic acid | 322.03 | 6 | 9 | 1.1 | 4 | 0 |
| Gallic acid | 170.02 | 4 | 5 | 0.7 | 1 | 0 |
| Gentisic acid | 154.03 | 3 | 4 | 1.6 | 1 |  |
| Isovanillic acid | 168.04 | 2 | 4 | 1.4 | 2 | 0 |
| Methyl gallate | 184.04 | 3 | 5 | 0.9 | 2 | 0 |
| Monogalloyl glucose | 332.07 | 7 | 10 | ND | 4 | 1 |
| Phthalic acid | 166.03 | 2 | 4 | 0.7 | 2 | 0 |
| *p*-Hydroxybenzoic acid | 138.03 | 2 | 3 | 1.6 | 1 | 0 |
| Protocatechuic acid | 154.03 | 3 | 4 | 1.1 | 1 | 0 |
| Syringaldehyde | 182.06 | 1 | 4 | 0 | 3 | 0 |
| Syringic acid | 198.05 | 2 | 5 | 1 | 3 | 0 |
| Vanillic acid | 168.04 | 2 | 4 | 1.4 | 2 | 0 |
| 3,5-Dimethoxy-4-[(trimethylsilyl)oxy]benzoic acid | 270.09 | 1 | 5 | ND | 5 | 0 |
| 4-Hydroxybenzaldehyde | 122.04 | 1 | 2 | 1.4 | 1 | 0 |
| Hydroxycinnamic acids | | | | | | |
| Caffeic acid | 180.04 | 3 | 4 | 1.2 | 2 | 0 |
| Caftaric acid | 312.04 | 5 | 9 | 0.1 | 7 | 0 |
| Chlorogenic acid | 354.10 | 6 | 9 | -0.4 | 5 | 1 |
| Cinnamic acid | 148.05 | 1 | 2 | 2.1 | 2 | 0 |
| Cryptochlorogenic acid | 354.10 | 6 | 9 | -0.4 | 5 | 1 |
| Ferulic acid | 194.06 | 2 | 4 | 1.5 | 3 | 0 |
| Linocaffein | 342.10 | 6 | 9 | -1.4 | 5 | 1 |
| Neochlorogenic acid | 354.10 | 6 | 9 | -0.4 | 5 | 1 |
| *p*-Coumaric acid | 164.05 | 2 | 3 | 1.5 | 2 | 0 |
| *p*-Coumaroylquinic acid | 338.10 | 5 | 8 | -0.1 | 5 | 0 |
| Rosmarinic acid | 360.08 | 5 | 8 | 2.4 | 7 | 0 |
| Sinapinic acid | 224.07 | 2 | 5 | 1.5 | 4 | 0 |
| 3,4-Di-O-caffeoylquinic acid | 516.13 | 7 | 12 | 1.5 | 9 | 3 |
| 3,5-Di-caffeoylquinic acid | 516.13 | 7 | 12 | 1.5 | 9 | 3 |
| Other phenolic acids | | | | | | |
| Carnosic acid | 332.20 | 3 | 4 | 4.9 | 2 | 0 |
| Homovanillic acid | 182.06 | 2 | 4 | 0.4 | 1 | 0 |
| Mandelic acid | 226.08 | 1 | 5 | 0.6 | 5 | 0 |
| *p*-Hydroxyphenylacetic acid | 152.05 | 2 | 3 | 0.8 | 2 | 0 |
| 3-(3,4-Dihydroxyphenyl)-2-hydroxypropanoic acid | 198.05 | 4 | 5 | -0.2 | 3 | 0 |
| 4-Hydroxyphenylacetic acid | 152.05 | 2 | 3 | 0.8 | 2 | 0 |
| Ellagic acid | 302.01 | 4 | 8 | 1.1 | 0 | 0 |
| Condensed tannins | | | | | | |
| Procyanidin B1 | 578.14 | 10 | 12 | 2.4 | 3 | 3 |
| Procyanidin B2 | 578.14 | 10 | 12 | 2.4 | 3 | 3 |
| Hydrolyzable tannins | | | | | | |
| Acutissimin | 1206.14 | 20 | 31 | 3 | 2 | 3 |
| bis-HHDP-glucose | 934.11 | 15 | 25 | 2.7 | 3 | 3 |
| Castalagin | 934.07 | 16 | 26 | 0.9 | 0 | 3 |
| Lambertianin C | 2748.24 | 43 | 76 | 6.5 | 13 | 4 |
| Peduculagin | 784.08 | 13 | 22 | 0.9 | 0 | 3 |
| Sanguiin H-6 | 1870.16 | 29 | 52 | 4.2 | 8 | 3 |
| Sanguiin H-10 | 1568.15 | 25 | 44 | 2.1 | 8 | 3 |
| Tannic acid | 1700.17 | 25 | 46 | 6.2 | 31 | 5 |
| Vescalagin | 934.07 | 16 | 26 | 0.9 | 0 | 3 |
| Ligands | | | | | | |
| Matairesinol | 358.14 | 2 | 6 | 3.3 | 6 | 0 |
| Secoisolariciresinol | 362.17 | 4 | 6 | 2.2 | 9 | 0 |
| Stilbenes | | | | | | |
| Resveratrol | 228.08 | 3 | 3 | 3.1 | 2 | 0 |
| Trans-epsilon-viniferin | 454.14 | 5 | 6 | 5.4 | 4 | 1 |
| Polydatin | 390.13 | 6 | 8 | 1.7 | 5 | 1 |
| Xanthone | | | | | | |
| Mangiferin | 422.08 | 8 | 11 | -0.4 | 2 | 2 |
| Mangiferin gallate | 574.10 | 10 | 15 | 0.2 | 5 | 3 |
| Isomangiferin | 422.08 | 8 | 11 | -0.4 | 2 | 2 |
| 1,3,5-Trihydroxy-4-prenylxanthone | 312.10 | 3 | 5 | 4.4 | 2 | 0 |
| Curcuminoids | | | | | | |
| Curcumin | 368.13 | 2 | 6 | 3.2 | 8 | 0 |
| Other polyphenols | | | | | | |
| Aesculin | 340.08 | 5 | 9 | -0.6 | 3 | 0 |
| Betaxanthin | 358.12 | 2 | 8 | 2.7 | 5 | 0 |
| Carnosol | 330.18 | 2 | 4 | 4.4 | 1 | 0 |
| Celecoxib | 381.08 | 1 | 7 | 3.4 | 3 | 0 |
| Coniferyl aldehyde | 178.06 | 1 | 3 | 1.5 | 3 | 0 |
| Gossypol | 518.19 | 6 | 8 | 6.9 | 5 | 2 |
| Hydroxytyrosol | 154.06 | 3 | 3 | -0.7 | 2 | 0 |
| Oleacein | 320.13 | 2 | 6 | 1.1 | 10 | 0 |
| Oleocanthal | 304.13 | 1 | 5 | 1.5 | 10 | 0 |
| Oleuropein | 540.18 | 6 | 13 | -0.4 | 11 | 2 |
| Pinoresinol | 358.14 | 2 | 6 | 2.3 | 4 | 0 |
| Tyrosol | 138.07 | 2 | 2 | 0.4 | 2 | 0 |
| 1-Acetoxypinoresinol | 416.15 | 2 | 8 | 1.8 | 6 | 0 |
| 2-Methyl-4-(1,1,3,3-tetramethylbutyl)- phenol | 220.18 | 1 | 1 | 5.3 | 3 | 1 |
| 3,5-Dimethoxyphenol | 154.06 | 1 | 3 | 1.6 | 2 | 0 |
| 3,5-Dimethoxy-4-[(trimethylsilyl)oxy]-benzaldehyde | 254.10 | 0 | 4 | NA | 5 | 0 |
| 6-Gingerol | 294.18 | 2 | 4 | 2.5 | 10 | 0 |
| 6-Shogaol | 276.17 | 1 | 3 | 3.7 | 9 | 0 |
| 8-Gingerol | 322.21 | 2 | 4 | 4.2 | 12 | 0 |

**Reference:**

Lipinski, C. A., Lombardo, F., Dominy, B. W., & Feeney, P. J. (2012). Experimental and computational approaches to estimate solubility and permeability in drug discovery and development settings. *Adv Drug Deliv Rev*, *64*, 4–17.
